# Young People’s Subjective Wellbeing in the Wake of the COVID-19 Pandemic: Evidence from a Representative Cohort Study in England

**DOI:** 10.1007/s11205-025-03776-7

**Published:** 2026-01-23

**Authors:** Jake Anders, Erica Holt-White

**Affiliations:** 1 UCL Centre for Education Policy & Equalising Opportunities, London, UK; 2 The Sutton Trust, London, UK

**Keywords:** COVID-19, Young people, Subjective wellbeing, Inequalities, Adverse life events, Social support

## Abstract

The COVID-19 pandemic and the disruption it has caused had substantial short-term effects on young people. These effects have been found to be highly unequal, exacerbating existing inequalities in society, including those associated with socio-economic status, gender and ethnicity. But, just as importantly, it is believed that they continue to cast a long shadow over some young people’s lives. In this paper we use data from the COVID Social Mobility & Opportunities study (COSMO) — a representative cohort study of over 13,000 young people in England aged 14–15 at pandemic onset whose education and post-16 transitions were acutely affected by the pandemic’s disruption through their remaining education and subsequent transitions — to highlight inequalities in young people’s subjective wellbeing and mental health in the wake of the pandemic. We document the substantial differences in subjective wellbeing — especially highlighting differences by gender — after adjusting for other demographic characteristics, self-reported levels of social support, and experience of adverse life events. We estimate how wellbeing differs by young people’s own perceptions of the ongoing impact of the pandemic: those who indicate an ongoing negative impact in their lives have substantially lower subjective wellbeing scores. Finally, we find a link between adverse life experiences during the pandemic and lower post-pandemic wellbeing, but do not find evidence that this is mediated by demographic characteristics or social support.

## Introduction

The COVID-19 pandemic and the disruption it caused had substantial short-term effects on young people’s lives around the world, with evidence of significant impacts on young people’s wellbeing and mental health (De France et al., 2022; Wolf & Schmitz, 2024). Young people in England, the focus of this paper, were no exception: extended periods in which in-person schooling was suspended (Anders, 2024) interrupted pupils’ learning (Jakubowski et al., 2025) and social lives (Kalenkoski & Pabilonia, 2024), with consequent rises in loneliness a clear symptom of this (Kung et al., 2023). This widespread disruption had widely documented short-term effects on young people’s wellbeing (e.g. Attwood & Jarrold, 2023; Banks & Xu, 2020; Neugebauer et al., 2024; Newlove-Delgado et al., 2021; Quintana-Domeque & Zeng, 2023), the magnitude of which was found to be linked with the intensity of lockdown restrictions (Owens et al., 2022), and the immediacy of which is reflected in wellbeing increasing and decreasing as restrictions tightened and eased (Creswell et al., 2021). A review by Kauhanen et al. (2023) summarised the international picture as “a longitudinal deterioration in symptoms for different mental health outcomes especially for adolescents and young people”.1

Existing analyses suggest that effects of the disruption were unequal, often exacerbating existing demographic inequalities in society, including those associated with socioeconomic status (e.g., Ravens-Sieberer et al., 2022), gender (e.g., Anders et al., 2023; Davillas & Jones, 2021), and ethnicity (e.g., Proto & Quintana-Domeque, 2021). Wolf and Schmitz (2024) finds that older adolescents were particularly affected, perhaps as these are such formative years for social relationships and critical years for education and subsequent transitions.

Variation in experiences and support during the pandemic has also been found to be important for young people’s wellbeing. Restrictions on social activities and the closure of schools reduced physical activity for some, which has been linked to worse mental health outcomes (Samji et al., 2022); other aspects of the pandemic are likely to have exacerbated the prevalence of adverse life events that previous studies have shown affect wellbeing (Cleland et al., 2016). Conversely, social support has been identified as a potential buffer to negative impacts (Demaray et al., 2005; Racine et al., 2021; Siedlecki et al., 2014) of such negative stressors. These highlight the potential importance of experiences and social support during the pandemic for young people’s wellbeing and, hence, the need to consider these in understanding differences in wellbeing.

While short-term impacts are important, we should be especially concerned if the impacts of the pandemic have continued to affect young people’s lives, including their subjective wellbeing, once restrictions ended. Concern was expressed from early in the pandemic that its negative effects on wellbeing would persist (Sonuga-Barke & Fearon, 2021), something that has been identified in some (Quintana-Domeque & Proto, 2022) but not all (Henseke & Schoon, 2025) studies of the general population. We explore the extent to which young people’s own perceptions of the ongoing impact of the pandemic on their wellbeing are associated with their post-pandemic subjective wellbeing, following in the spirit of research that seeks to understand the informational value of individuals’ own assessments of their situation (e.g. Fernandez-Urbano & Samuel, 2024 on perceived coping with the context of the pandemic).

Moreover, we should care about inequalities in young people’s wellbeing whether or not these are (entirely) due to the pandemic. Indeed, we may be more concerned about the persistence of inequalities that pre-date the pandemic — including in gender (Yoon et al., 2023) and socioeconomic status (Verhulst & Tiemeier, 2020) — for which there is no particular reason to expect them to subside. As such, taking stock of the current situation is important in its own right given correlation between subjective wellbeing and later economic and wider outcomes (Deaton, 2008; Lyubomirsky et al., 2005).

In our analyses of these issues, we are guided by Social Production Function (SPF) theory (Ormel et al., 1999), which enumerates five components contributing to subjective well-being: stimulation, comfort, status, behavioural confirmation, and affection. While this study does not engage individually with all five factors, SPF nevertheless provides a helpful framework, including in distinguishing between long-term factors such as status, linked with socioeconomic and demographic characteristics, and more acute potential impacts of changes to stimulation, comfort and affection presented by the disruption of the pandemic and specific events during its course. Furthermore, the potential buffering role of social support can be seen as integral to the SPF components of status and affection. Focussing particularly on the context of the COVID-19 pandemic, Chesters (2025) posit that its restrictions may have negatively affected young people’s access to affection, when not able to spend time with friends and extended family; stimulation, due to restrictions on activities; comfort, both material through potential financial distress, and emotional through adverse life events; and behavioural confirmation, through the disruption to routines and societal expectations.

Using data from the COVID Social Mobility & Opportunities study (COSMO) — a representative cohort study of over 13,000 young people in England aged 14–15 at pandemic onset whose education and post-16 transitions were acutely affected by the pandemic’s disruption through their remaining education and subsequent transitions — we provide new evidence regarding these issues, specifically through the following research aims:

1. to estimate differences in post-pandemic wellbeing among this cohort by demographic characteristics;
2. to validate and quantify the informational value of young people’s own perceptions of the impact of the pandemic on their wellbeing, along with the extent to which this may be explained by differences in social support and;
3. to explore the role of adverse experiences during the pandemic in explaining differences in post-pandemic wellbeing, again accounting for the potential importance of differences in social support.

The paper proceeds as follows. In Sect. 2, we describe the data that we use, the steps taken to prepare it for analysis, and conduct descriptive analyses to provide initial evidence on our research aims. In Sect. 3, we describe our use of regression modelling, presenting results in Sect. 4. Finally, we discuss our findings and conclude in Sect. 5, noting implications for policy and practice.

## Data and Descriptive Analyses

We use data from the COVID Social Mobility & Opportunities study (COSMO), a longitudinal cohort study following a representative sample of young people (and their parents) who were in Year 10 (i.e., aged 14–15) at pandemic onset (March 2020), who participated at both waves 1 (Anders et al., 2024a), carried out October 2021–March 2022 (ages 16–17), and 2 (Anders et al., 2024b), carried out October 2022–March 2023 (ages 17–18). In both cases the majority of interviews were carried out within the first two months of fieldwork; we also control for month of interview in our regression models (further details below).

COSMO has a clustered and stratified design with oversampling of those from smaller (e.g., ethnic minorities), more disadvantaged and harder to reach demographic groups to improve statistical power when exploring inequalities between such groups. Furthermore, there was initial non-response and attrition between the first two waves. As such, it is important to account for the deliberate and modelled sample disproportionalities, as well as implications of clustering and stratification for statistical inference. We take these features into account in analyses using study-provided clustering and stratification variables, and design and non-response weights (Adali et al., 2022, 2023).

To ensure consistency across analyses, we restrict our sample to those with valid data on the key variables for our analyses. This includes the primary outcome of self-reported wellbeing score, along with key predictors and demographic variables. However, we are mindful of the potential implications of sample selection caused by complete case analysis. We robustness check our findings in Sect. 9, re-running our core analyses having only restricted the sample based on the primary outcome (wellbeing score) and the main predictors (impact of pandemic on mental health and adverse life events reporting) and multiply imputing across 10 datasets all other predictors using a highly flexible classification and regression tree approach (Lumley, 2019; van Buuren & Groothuis-Oudshoorn, 2025).

### Subjective Wellbeing

To measure self-reported wellbeing, we use the UK Office for National Statistics’ official measure of life satisfaction (Office for National Statistics, 2018), which is widely recognised as an important dimension of subjective wellbeing (Petersen et al., 2022). This asks participants to respond to the prompt “Overall, how satisfied are you with your life nowadays?” on a scale ranging from 0 “Not at all satisfied” to 10 “Completely satisfied”. This measure has been used in national UK surveys since 2011 and increasing numbers of academic studies, hence providing a useful benchmark for this concept in UK-based surveys. This measure is found to be a reliable measure of subjective wellbeing in young people (Levin & Currie, 2014), performing as well as the more in-depth Satisfaction with Life Scale (Jovanović, 2016), for example, although we do recognise that it will not capture all dimensions of wellbeing (Ruggeri et al., 2020). It is also worth noting that, while they are distinct constructs, a clear correlation between lower wellbeing and increased risk of poor mental health (Lombardo et al., 2018).

As COSMO was established in response to the pandemic, there are no pre-pandemic baseline measures. As such, we emphasise that our estimates of differences are between individuals all of whom have experienced the pandemic, but experienced it differently, rather than between their current situation and a counterfactual in which the pandemic did not happen. Others have used survey experiment methods to attempt to get closer to such a counterfactual (Andreoli et al., 2024), or pre-existing longitudinal studies to explore change in mental health across the pandemic period (Henseke & Schoon, 2025).

**Fig. 1:**
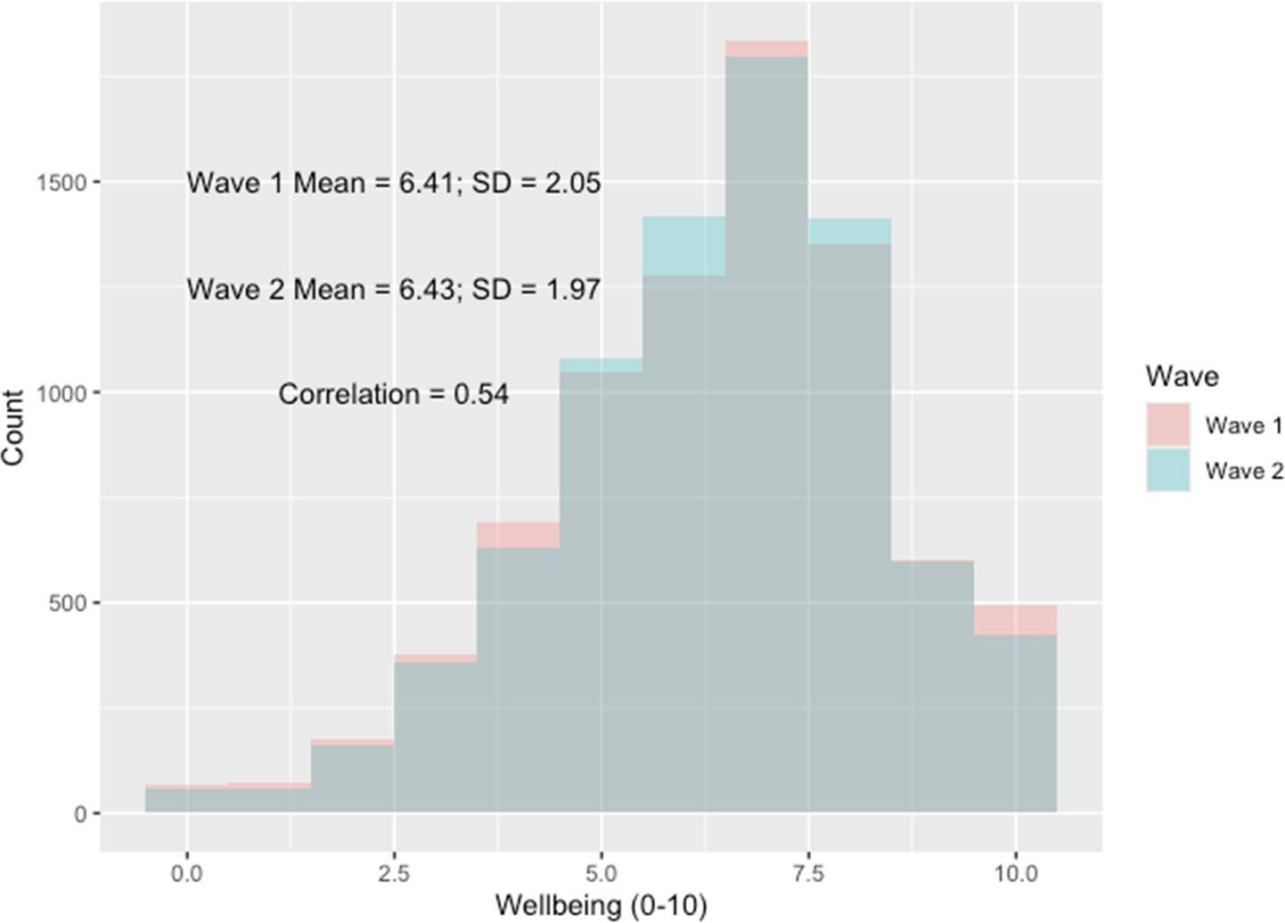
Histogram of distribution of subjective wellbeing in Wave 1 and 2. Notes: Histogram weighted for survey design and non-response

We have measures of wellbeing from two post-pandemic waves and use these to explore evidence of change in wellbeing between the two waves both overall, and between sub-groups of the data where this might be expected. We plot the overall distribution of reported wellbeing in both Waves 1 (age 16/17) and 2 (age 17/18) in Fig. 1.

Young people report a mean wellbeing score of 6.41 in Wave 1 and 6.43 at Wave 2, with the standard deviation declining slightly from 2.05 to 1.97. These are not particularly substantial changes, providing little evidence of change between these two post-pandemic time points. However, in interpreting this (lack of) aggregate change, we must be mindful of this cohort’s wider context.

One interpretation would be that, as we know there was a decline in mental health and wellbeing among young people at the onset of the pandemic and its restrictions (Newlove-Delgado et al., 2021), we would hope to see an upward trajectory in wellbeing in subsequent years to be confident of a ‘bounce back’, with this lack of change suggesting a plateau at a lower level than before the pandemic. That could be the case. A finding of minimal change is consistent with the findings of Henseke et al. (2022) (albeit for a wider age range of young people aged 16–29). Similarly, the UK Office for National Statistics’ annual population survey also suggests that life satisfaction has not returned to pre-pandemic levels in the general population (Office for National Statistics, 2023).

Fundamentally, using these data alone we are unable to adjudicate between multiple potential plausible scenarios. Others, using a wider range of datasets are better placed to do so. For example, Henseke and Schoon (2025) suggest that young people’s wellbeing may have already returned to pre-pandemic levels, thus explaining a lack of trend for this reason. These findings would also be consistent with an upward post-pandemic trend being cancelled out by a countervailing age effect (for example) implied by the wider literature on life course wellbeing (Blanchflower, 2021).

However, this is not our paper’s focus. Aggregate stability does not mean that there are not individual-level differences or differential change in wellbeing. The correlation between the reported measures in Waves 1 and 2 is 0.54. While some of this likely reflects natural fluctuation in young people’s wellbeing due to daily idiosyncratic shocks, it provides a basis to explore evidence of systematic difference in change between the two waves, along with the differences in levels at each wave.

### Social Support

Social support is concerned with the extent to which an individual is, or perceives they are, “cared for, esteemed, and valued by people in [their] social network” (p. 691, Demaray et al., 2005). As such, it directly relates to the SPF framework, specifically status and affection. As such, we anticipate that individuals with greater social support will have higher levels of wellbeing (Li et al., 2021; Magson et al., 2021; Siedlecki et al., 2014). Furthermore, because of the potential for substitution between components of the SPF in the production of wellbeing (Ormel et al., 1999), we also anticipate social support buffering shocks to other aspects; this has been observed empirically with social support buffering shocks to wellbeing in the face of adversity (Aksoy et al., 2024; Kearns et al., 2015; McMahon et al., 2023).

To capture this factor, we use the social provisions scale (Cutrona & Russell, 2018), specifically a shortened three-item variant available in COSMO in which young people are asked to respond (using the categories “Not true”, “Partly true” or “Very true”) to the statements:

1. I have family and friends who help me feel safe, secure and happy.
2. There is someone I trust whom I would turn to for advice if I were having problems.
3. There is no one I feel close to [Negatively coded].

**Fig. 2:**
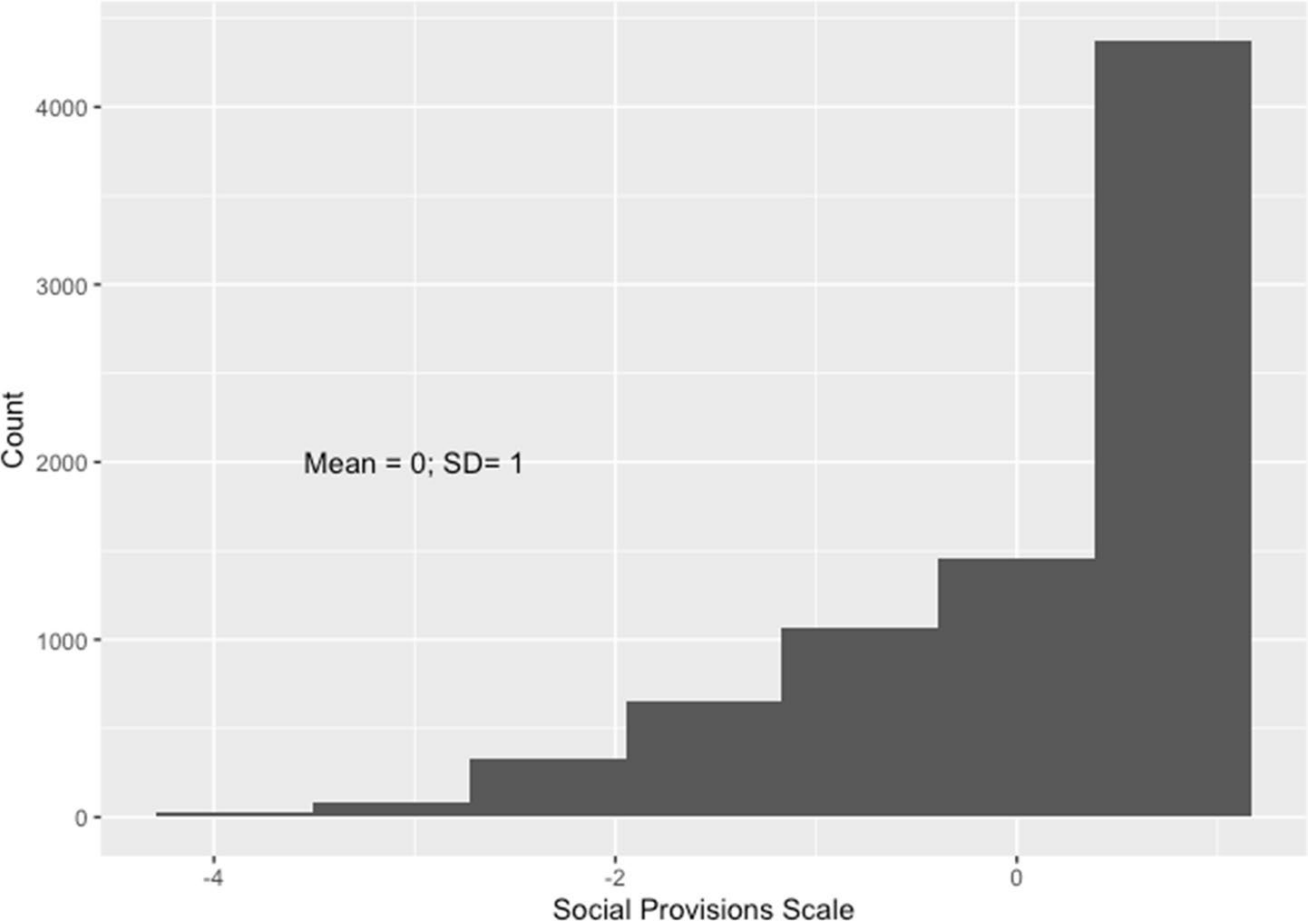
Distribution of social provisions scale. Notes: Distribution of social provisions scale. The scale is standardised to have mean 0 and standard deviation 1 in the analysis sample. Weighted for survey design and non-response

Following standard practice, we sum over the values of the three items and standardise the resulting variable (mean zero; standard deviation one) for the purposes of interpretation. We plot the distribution of the social provisions scale in Fig. 2. There is some evidence of a ceiling effect — most respondents score the maximum of 6 — but with a decent spread below this. We use this as a continuous measure in our analyses.

### Demographic Characteristics

The impact of the pandemic on young people’s wellbeing differed depending upon their demographic characteristics (e.g., Anders et al., 2023). Both to estimate differences between young people based on these characteristics, and to control for these measures in other analyses, we make use of the rich set of demographic measures collected in COSMO. Specifically, we construct the following measures of demographic characteristics.

- *Gender*: There are longstanding concerns about differences in wellbeing by gender (Yoon et al., 2023), which have only been exacerbated by the pandemic (Davillas & Jones, 2021). We use young people’s reported gender at either wave (giving precedence to the subsequent reports if they differ) and group cohort members into ‘female’, ‘male’ and ‘non-binary+’, where the final category is a combination of those who explicitly report being non-binary or choose to identify in any other way (since these other groups are too small for analysis).
- *Ethnicity*: As with gender, our measure is based on self-reports at either wave (where a subsequent report is given precedence if they differ), young people are grouped into ‘White’, ‘Mixed’, ‘Black’, ‘Asian’ and ‘Other’. While these categories are broad, they are chosen for consistency with the UK’s major ethnic group classifications while avoiding groups that are too small for analysis purposes.
- *Parental education*: Generally viewed as a core component of socioeconomic status, which may affect wellbeing through the status component of the SPF (Ormel et al., 1999), we construct our measure using the highest level of education reported by either parent at either wave, grouping parents into ‘Graduate’, ‘Below Graduate’ and ‘No Quals’.
- *Housing tenure*: Housing tenure is another component of socioeconomic status, hence with potential implications for young people’s wellbeing. Our measure is based on parental reports at either wave (giving precedence to the subsequent if they differ), grouping families into those who own their home (either with a mortgage or outright; ‘Own House’) and all others (predominantly social and private renting; ‘Other’).
- *Area deprivation*: We also include an area-based measure of deprivation of participants’ homes, both as a correlate of socioeconomic stats due to residential sorting and given more direct implications this can have for potentially wellbeing-enhancing amenities. COSMO provides decile groups of the UK’s Income Deprivation Affecting Children Index (IDACI), constructed at the ‘lower-layer super-output area’ (the smallest geographical areas in UK statistical geography, containing an average population of 1,500).

To allow exploration of differences in wellbeing by socioeconomic status (SES) in a simple way, we create a combined index of SES (with mean 0 and standard deviation 0 in our analysis sample) across our measures of parental education, housing tenure and home neighbourhood deprivation. We describe how we do this and demonstrate that it captures the underlying SES measures on which it is based in Sect. 6.1.

Having constructed this set of measures, we report the prevalence of demographics in our cohort along with mean levels of self-reported wellbeing by these categories at Wave 1, Wave 2, and mean difference between the two in Table 1.

**Table 1:** Mean subjective wellbeing score by demographic characteristics

Characteristic	*N*	Prevalence (%)	Wave 1	Wave 2	Difference
Overall	7,723		6.41	6.43	0.017
Gender					
Male	3,475	50	6.76	6.76	0.007
Female	4,030	48	6.13	6.15	0.021
Non-Binary+	218	2.6	4.90	5.04	0.136
Ethnicity					
White	4,877	77	6.43	6.44	0.014
Mixed	477	5.7	6.09	6.09	−0.008
Black	1,503	10	6.51	6.48	−0.030
Asian	684	5.0	6.34	6.43	0.094
Other	182	2.2	6.44	6.64	0.201
Parental Education					
Graduate	3,807	55	6.48	6.45	−0.024
Below Graduate	2,962	36	6.35	6.37	0.024
No Quals	871	7.6	6.25	6.54	0.286
Unknown	83	0.8	6.42	6.37	−0.049
Housing Tenure					
Own House	4,224	65	6.50	6.54	0.037
Other	3,499	35	6.24	6.22	−0.020
Unknown	0	0			
IDACI Quintile Group					
1 (High Deprivation)	2,306	22	6.24	6.23	−0.007
2	1,678	19	6.39	6.42	0.032
3	1,351	19	6.34	6.46	0.118
4	1,231	20	6.54	6.56	0.023
5 (Low Deprivation)	1,157	20	6.56	6.49	−0.072
SES Quintile Groups					
1 (Low SES)	2,257	20	6.26	6.26	0.005
2	1,770	20	6.28	6.37	0.088
3	1,405	20	6.42	6.40	−0.019
4	1,266	21	6.53	6.53	0.006
5 (High SES)	1,025	19	6.58	6.58	0.002

Reporting means except where otherwise specified. All estimates are weighted and account for the complex survey design. The difference is calculated as Wave 2 - Wave 1

50% of the sample are male, 48% are female and 2.6% are non-binary or report in another way. Average reported wellbeing differs substantially between these groups with boys (6.76 in Wave 1) reporting higher levels of wellbeing than girls (6.13). This is consistent with existing work on inequalities in young people’s wellbeing (e.g. Anders et al., 2023, Davillas & Jones, 2021), both before the pandemic and as a result of its impact. Non-binary + young people report lower levels of wellbeing still than girls, although there is evidence of an increase for this group between Waves 1 and 2; we should be mindful, however, of the smaller sample size for this group.

By ethnicity, the highest levels of reported wellbeing are for Black young people (6.51 in Wave 1), followed by White young people (6.43), with the lowest among young people who reported a Mixed ethnicity. These differences are small and, other than the small group of young people placed into the Other category, there is little evidence of change over time.

There is a broadly consistent gradient in wellbeing across our quintile groups of socioeconomic status, from 6.26 to 6.58 (both for Wave 1 but with a similar picture in Wave 2). Again, these appear to be rather small differences and there is no evidence of consistent change between the two waves.

Overall, this initial analysis highlights gender as the most important demographic difference in wellbeing for this sample of young people in England.

### Perceived Ongoing Impact

Next, we seek to quantify differences in young people’s wellbeing by their own perceptions of the ongoing impact of the pandemic. This takes seriously young people’s own reports of the ongoing impact of the pandemic on their wellbeing. To capture these perceptions, we use a question asked to young people at the second wave of COSMO, asking “Would you say the pandemic is still having an effect on [your mental wellbeing], whether positive or negative?” If they agree with this question then they are subsequently asked to distinguish whether this impact is positive, negative or they don’t know.

Table 2 shows that 64% of young people report that the pandemic is continuing to have an impact on their mental wellbeing, with 32% of these reporting that this impact is negative. Perhaps unsurprisingly, much smaller proportion of young people report that the ongoing impact is positive (2%) or that they don’t know if the impact is positive or negative (2%).

**Table 2:** Mean subjective wellbeing score by whether and how the pandemic continues to affect mental wellbeing

Variable, *N* = 7723	No (64%)^1^	Negative (32%)^1^	Don’t know (2%)^1^	Positive (2%)^1^	Overall (100%)^1^	*p*-value^2^
Wave 1	6.81	5.62	6.37	6.40	6.41	< 0.001
Wave 2	6.91	5.46	6.29	6.48	6.43	< 0.001
Difference	0.11	−0.16	−0.08	0.08	0.02	< 0.001

^1^Mean

^2^Design-based KruskalWallis test

All estimates are weighted and account for the complex survey design. The difference is calculated asWave 2 - Wave 1

Those who report no impact of the pandemic on their mental wellbeing have the highest self-reported wellbeing (6.81 in Wave 1; 6.91 in Wave 2), while those who report that it had a negative impact on their mental wellbeing report the lowest (5.62 in Wave 1; 5.46 in Wave 2). Those who say it is still having an impact but that it is positive, or that they don’t know if it is positive or negative, report somewhere between the other two groups but, as noted, these are a very small proportion of the sample.

These groups are also distinguished by changes in reported wellbeing between Waves 1 and 2. Those who report that the pandemic is continuing to have a negative impact on their mental wellbeing do, indeed, report a decline in wellbeing (−0.16) between the two waves, while those who report that it has had no impact (0.11) or that it is having a positive impact report an increase (0.08). Those who report that it is still having an impact but that they don’t know if it is positive or negative report a slight decline (−0.08). These last two groups are small, so these estimates should be treated with caution. In subsequent analyses we combine these two groups with the group who report no impact, for an overall comparison of those who report an ongoing negative impact with the rest of the sample.

### Adverse Life Events

Finally, we explore whether subjective wellbeing is associated with experiencing adverse life events during the COVID-19 pandemic. We report details of the construction of this measure in Sect. 6.2, creating a composite index of adverse life events using polychoric principal component analysis (PCA) of the ten adverse life events available in the data and dividing the sample into tertile groups based on the resulting index.

We find that mean wellbeing score differs by experience of such events (Table 3). Wellbeing is lower for those who experience a higher prevalence of adverse life events, ranging from 7.04 for those in the fewer adverse life events tertile group to 5.67 for those in the more adverse life events tertile group. This pattern is consistent across Waves 1 and 2, but there is no significant evidence of difference in the patterns of change over time.

**Table 3:** Mean subjective wellbeing score by experience of adverse life events reported since onset of pandemic

Variable, *N* = 7723	Fewer (36%)^1^	Average (30%)^1^	More (33%)^1^	Overall (100%)^1^	*p*-value^2^
Wave 1	7.04	6.46	5.67	6.41	< 0.001
Wave 2	7.02	6.47	5.75	6.43	< 0.001
Difference	−0.03	0.00	0.08	0.02	0.2

^1^Mean

^2^Design-based KruskalWallis test

All estimates are weighted and account for the complex survey design

However, as with all our descriptive analyses, we are mindful that there is the potential for differences in socioeconomic and demographic characteristics between by experience of adverse life events. For this reason, as well as for our other analyses, we use regression modelling to unpack these findings further.

## Analytical Approach

To extend our descriptive analyses and, hence, provide a more nuanced understanding of the factors associated with young people’s wellbeing since the pandemic, we use regression modelling. All analyses are carried out using R (R Core Team, 2024), with the survey package (Lumley et al., 2024) used to account for the complex survey design of the data, including design and non-response weights, and adjustments to statistical inference due to stratification and clustering of the sample.

We break this section into three sub-sections, aligned with the research aims in this paper: demographic differences in subjective wellbeing; the importance of perceived ongoing impact of the pandemic; and the importance of adverse life events during the pandemic.

### Demographic Differences in Subjective Wellbeing

First, we use linear regression models to explore differences in young people’s wellbeing. These models all take the form

1 $$\begin{array}{c}\:LifeSat_{it}={\beta\:}_0+\beta\:{'\:}_1SES_i+\\\beta\:{'\:}_2Gender_i+\beta\:{'\:}_3Ethnicity_i\\+X{'\:}_i+{\epsilon\:}_{it}\:\end{array}$$

where $\:LifeSat$ is wellbeing score for person $\:i$ at time $\:t$, $\:SES$ is a vector of binary variables for quintile groups of SES (leaving the highest SES quintile group as the omitted category), $\:Gender$ is a vector of binary variables for gender (Female and Non-binary+, leaving Male as the omitted category), $\:Ethnicity$ is a vector of binary variables for ethnicity (Asian, Black, Mixed, Other, leaving the largest category, White, omitted as the baseline), $\:X$ is a vector of covariates varying between model specifications discussed below, and $\:\epsilon\:$ is the error term. We estimate these models separately for each time point of the survey, and then again for Wave 2 with an additional covariate of Wave 1 wellbeing score to provide estimates of difference adjusting for Wave 1 wellbeing as a baseline.

We estimate a series of models summarised in Table 4, beginning with simple models including gender (L1), ethnicity (L2), and SES (L3) entered separately, replicating the descriptive analyses and unconditional estimates of differences in wellbeing reported in Table 1. Next, we include all three demographic characteristics at the same time in L4, along with the addition of a month of interview variable to allow for potential confounding due to the timing of the survey. This model, hence, provides estimates of demographic differences in wellbeing, conditional on the other demographic characteristics included. We then explore potential intersectional differences in wellbeing between demographics in L5 (Codiroli Mcmaster & Cook, 2019) by including a full set of interaction terms between our SES, gender and ethnicity variables.

**Table 4:** Model specifications for regression analysis of subjective wellbeing

Variable	L1	L2	L3	L4	L5	L6	L7	L8
Gender	Included			Included	Interacted w/Ethnicity and SES	Included	Interacted w/Social Support	Included
Ethnicity		Included		Included	Interacted w/Gender and SES	Included	Interacted w/Social Support	Included
SES			Included	Included	Interacted w/Gender and Ethnicity	Included	Interacted w/Social Support	Included
Social Support						Included	Interacted w/Gender, Ethnicity and SES	Included
Adverse Events								Included

L1-L7 refer to the model number. SES = Socioeconomic status

Next, motivated by understanding the potential importance of social support in explaining these differences, we add social provisions score in L6. Differences between the coefficients on our demographic characteristics between L4 and L6 will, hence, provide information on the extent to which differences in social support explain the unadjusted differences.

L7 explores whether the importance of social support varies by demographic characteristics. As with L5, we include interaction terms, this time between our demographic characteristics and the two social support measures to allow for the moderation of the relationship between these measures and wellbeing.

Finally, L8 explores the importance of adverse life events in explaining demographic differences in wellbeing. We include the adverse life events index in this model, along with the demographic characteristics and social support measures. Comparing coefficients on the demographic characteristics in L6 and L8 hence provides information on the extent to which differences in adverse life events may explain demographic differences in wellbeing. We do not model the interaction between adverse life events and demographic characteristics at this point as we explore this in a subsequent section.

### Importance of Perceived Impact of the Pandemic on Wellbeing

In this section, we again use linear regression models to estimate differences in subjective wellbeing. However, this time we focus on differences explained by young people’s perceptions of the ongoing impact of the pandemic on their life. The models take the form:

2 $$\:LifeSat_{it}={\beta\:}_0+\beta\:{'\:}_1PandemicImpactPercep_i+X{'\:}_i+{\epsilon\:}_{it}\:\:\\$$

where definitions are per Eq. 1, and $\:PandemicImpactPercep$ is a binary variable indicating that person $\:i$ reports that the pandemic is continuing to have a negative impact on their life. We, again, estimate separate models for each time point, as well as for Wave 2 adjusting for Wave 1.

The series of models is summarised in Table 5, with the first model (P1) replicating our descriptive findings by including no additional covariates, meaning the coefficient on $\:PandemicImpactPercep$ reports the difference between those who report that the pandemic had a negative impact on their mental wellbeing and the rest of the cohort.

**Table 5:** Model specifications for regression analysis of subjective wellbeing

Variable	P1	P2	P3	P4
Perceived Impact	Included	Included	Included	Interacted with Demographics, SES and Social Support
Demographics		Included	Included	Interacted with Perceived Impact
SES		Included	Included	Interacted with Perceived Impact
Social Support			Included	Interacted with Perceived Impact

P1-P4 refer to the model number. SES = Socioeconomic status

Next, in P2, we include demographic (gender, ethnicity), methodological (month of survey) and socioeconomic status (parental education, housing tenure, and area-level deprivation) covariates. We do this, rather than including combined SES quintile groups, now that we are not trying to interpret an overall SES association but rather adjust for these as flexibly as possible. Our focal coefficient from this model thus estimates the difference in wellbeing associated with a continuing negative perception of the pandemic on wellbeing among those with similar socio-demographic characteristics.

We then explore the extent to which differences in wellbeing associated with a negative perceived impact of the pandemic are explained by social support. In P3, we add social provisions score and compare the estimate on our focal variable coefficient between models P2 and P3.

Finally, in P4, we explore evidence of variation in the difference in wellbeing associated with a negative perceived impact of the pandemic by demographic and social support measures. We do this by including a full set of interaction terms between our focal variable and the socio-demographic and social support variables in P3.

### Importance of Adverse Life Events During the Pandemic

For our final aim, we explore the importance of adverse life events during the pandemic in explaining young people’s wellbeing post-pandemic.

To do so, we use linear regression models to explore the extent to which differences in self-reported wellbeing depend on the adverse life experiences they faced, including conditional on their perception of the impact of the pandemic on their wellbeing. The models used for this purpose take the form:

3 $$\:LifeSat_{it}={\beta\:}_0+\beta\:{'\:}_1TAdverseEventIndex_i+X{'\:}_i+{\epsilon\:}_{it}$$

where definitions are per Eq. 1, and $\:TAdverseEventIndex$ is a vector of binary variables indicating person $\:i$’s location in the distribution of the adverse life event index (more and average, leaving fewer as a baseline). We, again, estimate separate models for each time point, as well as for Wave 2 adjusting for Wave 1. When modelling Wave 1 wellbeing, a variant of our events index is used based on Wave 1 event reports only.

Our models are summarised in Table 6, with the first model (E1) again replicating our descriptive findings by including only the tertile groups of the adverse life events index. In preliminary work, we explored alternative modelling approaches including using the index as a continuous variable or including the individual adverse life events, as listed in Sect. 6.2. Including tertile groups provided the most interpretable results without substantively affecting model fit.

**Table 6:** Model specifications for regression analysis of subjective wellbeing by life events

Variable	E1	E2	E3	E4	E5
Adverse Events	Included	Included	Included	Included	Interacted with Demographics, SES, Social Support and Perceived Impact
Demographics		Included	Included	Included	Interacted with Adverse Events
SES		Included	Included	Included	Interacted with Adverse Events
Social Support			Included	Included	Interacted with Adverse Events
Perceived Impact				Included	Interacted with Adverse Events

E1-E5 refer to the model number. SES = Socioeconomic status

Next, in E2, we add demographic characteristics (gender, ethnicity), socioeconomic status (parental education, housing tenure, and area-level deprivation), and month of survey. This model thus estimates the difference in wellbeing associated with greater experiences of adverse life events during the pandemic among those with similar socio-demographic characteristics, as well how much distribution of events across socio-demographic groups explains wellbeing differences.

We then explore how much differences in wellbeing associated with adverse life events are explained by social support. In E3, we add social provisions score and compare the estimate on our focal variable between models E2 and E3. This is very similar to model L6, but with adverse life events as our focus so these are entered using the tertile groups to aid interpretation.

Next, we include perceived ongoing impact of the pandemic (focal variable in the previous section). As we hypothesise that at least some of the formation of ongoing perceptions of negative impact from the pandemic is due to experience of adverse events, this model (E4) is not a reliable guide to the association between adverse events and wellbeing: including the perception variable is over-controlling. However, the model is useful in comparison with P3 in demonstrating how much of the difference in wellbeing associated with a negative perception of the ongoing impact of the pandemic on wellbeing is explained by experience of adverse life events.

Finally, analogously to previous sections, we include interactions of our focal variables (experience of adverse life events) with our socio-demographic and social support measures in model E5.

## Results

In this section, we report results from the regression models outlined in the previous section, beginning with demographic differences in wellbeing Sect. 4.1, then the importance of perceived ongoing impact of the pandemic Sect. 4.2 and, finally, the importance of adverse life events during the pandemic Sect. 4.3. We report our results graphically, focussing attention on the estimates pertinent to addressing our research aims and allowing for easy comparison across models. We provide full regression tables of the results for each model, which are included in Sect. 7 for reference.

### Demographic Differences in Subjective Wellbeing

**Fig. 3:**
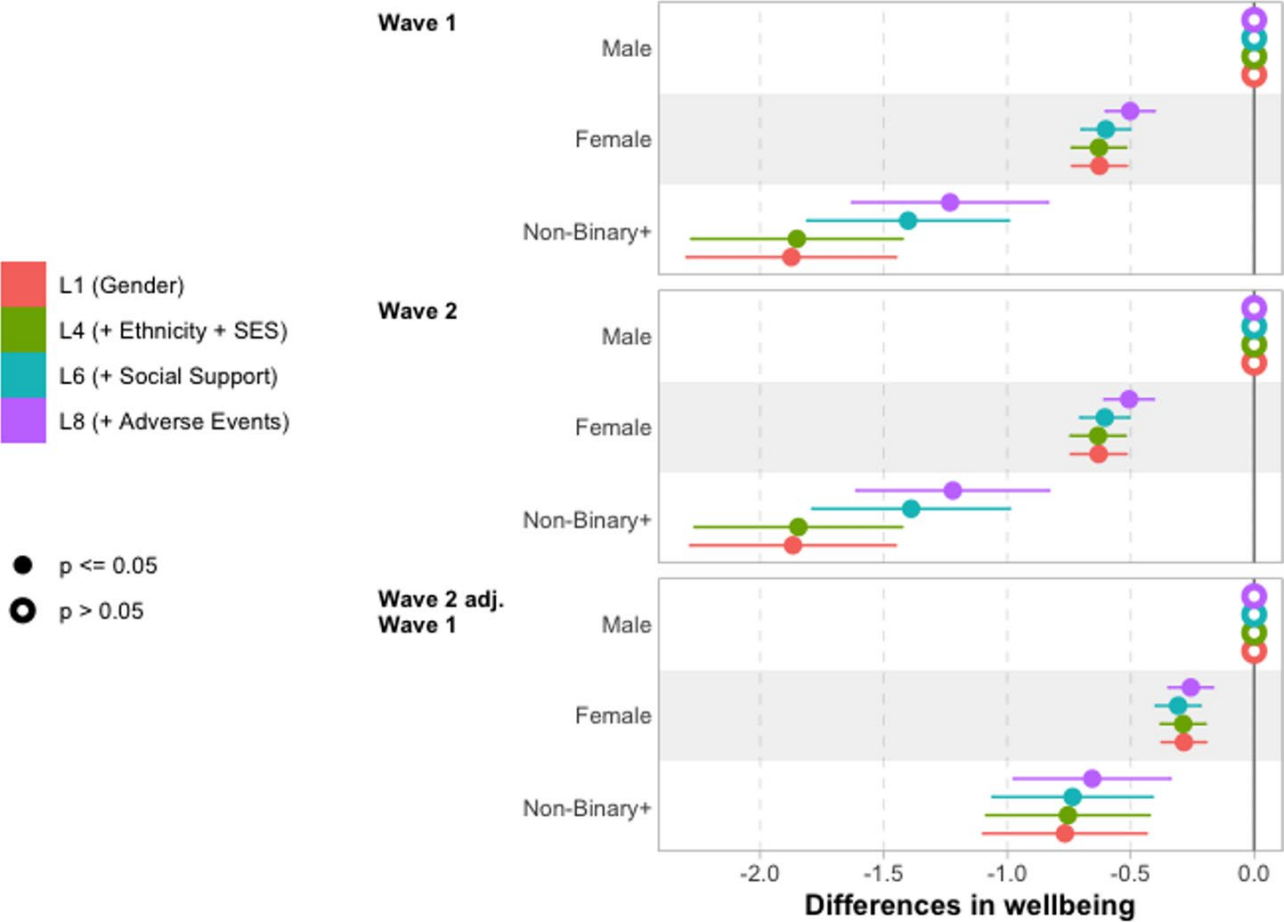
Differences in wellbeing by gender **Notes**: Reporting coefficients from underlying regression models reported in Tables 9 and 10, and Table 11

First, we explore overall differences in wellbeing, through the series of models summarised in Table 4. The core results are plotted in Fig. 3 for gender, Fig. 9 for ethnicity, and Fig. 10 for SES. In each case, results are presented for Wave 1, Wave 2, and Wave 2 adjusted for Wave 1, with the discussion starting out with Wave 1 in each case, before discussing notable differences in Wave 2, or Wave 2 adjusted for Wave 1. Full results tables for these models are reported in Sect. 7: Table 9 for Wave 1, Table 10 for Wave 2, and Table 11 for Wave 2 adjusted for Wave 1.

In the case of gender (Fig. 3), we essentially replicate the descriptive findings (Table 1) in L1, finding that girls’ wellbeing is 0.63 points lower than for boys, and a larger reduction for those grouped as non-binary + where the reduction is 1.9 points compared to boys. There is essentially no change when we adjust for ethnicity and SES in L4, with the differences remaining 0.63 points for girls and 1.9 points for non-binary + young people.

Part of the difference in wellbeing among non-binary + young people is explained by variation in social support: when including social provisions in L6 the difference reduces to 1.4 points compared to boys. This makes a similar difference at Wave 2, but no difference for girls at any wave, nor for non-binary + youth when considering Wave 2 wellbeing adjusted for Wave 1 wellbeing.

A small part of the remaining difference is explained by experiences of adverse life events, reducing to 1.2 for non-binary + young people and to 0.5 for girls, although the difference between L6 and L8 is not statistically significant for the non-binary + group, nor quite statistically significant at the 5% level for girls.

We do not find consistent differences in wellbeing by ethnicity or gender after adjusting for covariates; reporting of these results may be found in Sect. 8.

### Perceived Continuing Impact of the Pandemic on Wellbeing

**Fig. 4:**
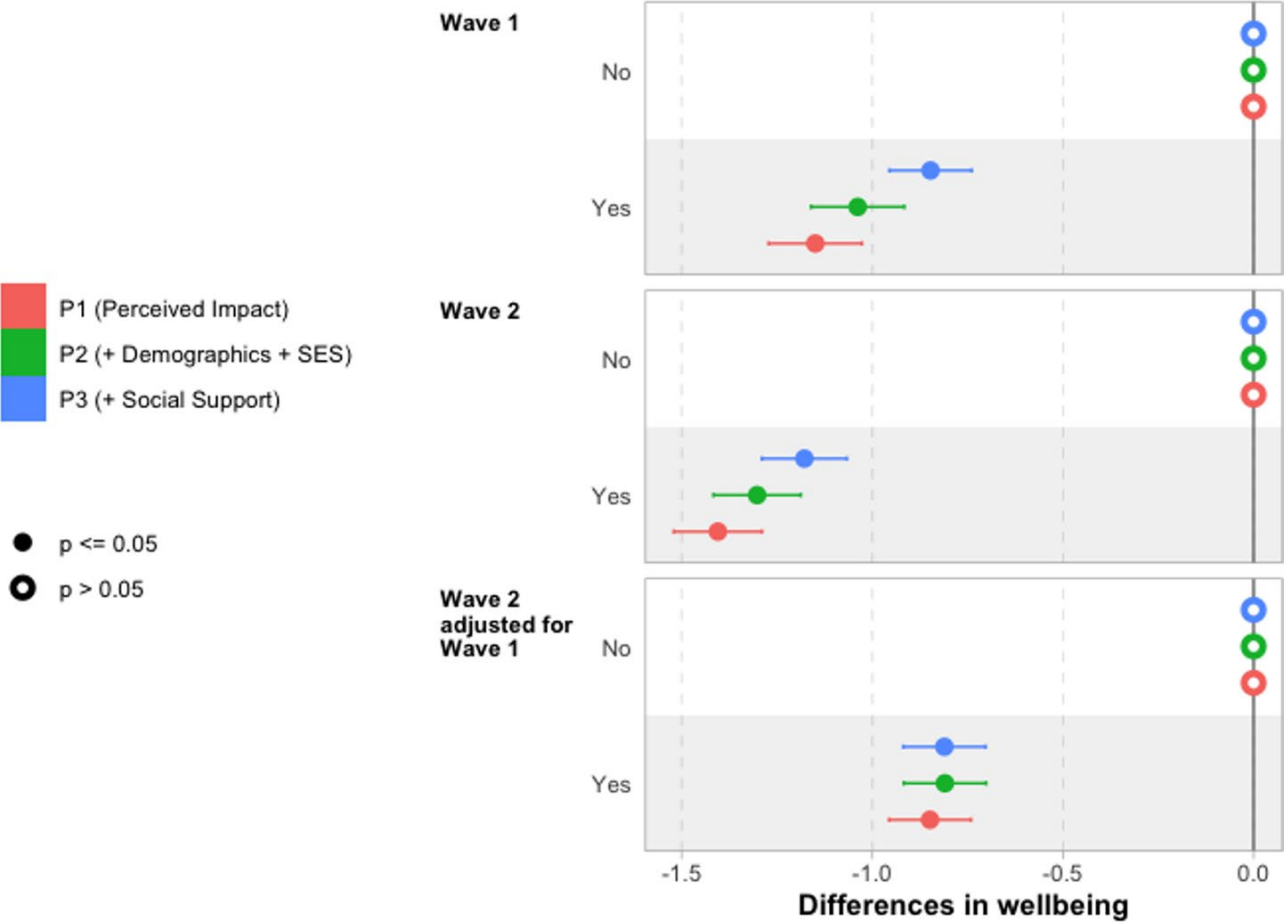
Differences in wellbeing by perceived continuing impact of pandemic on wellbeing Notes: Reporting coefficients from underlying regression models reported in Tables 12 and 13, and Table 14.

Next, we discuss differences in wellbeing by perceived continuing impact of the pandemic using the models summarised in Table 5. Core results are plotted in Fig. 4. Full tables of results for these models are reported in Sect. 7, Table 12 (Wave 1), Table 13 (Wave 2) and Table 14. (Wave 2 adjusting for Wave 1).

Results from unconditional model P1 indicate that young people who perceive a negative continuing impact of the pandemic on their wellbeing report 1.1 points lower wellbeing score than those who do not perceive such an impact. Perhaps surprisingly, given the greater time that has elapsed since the pandemic, this difference is larger at Wave 2, with a 1.4-point difference between these two groups. However, we should recall that the report of a negative continuing impact of the pandemic is collected at Wave 2, so may reflect this being more contemporary with the report.

A fairly small part of the difference in wellbeing score is explained by inclusion of demographic characteristics (in P2) and social support (in P3). The differences are reduced to 0.85 points and 1.2 points at Wave 1 and Wave 2, respectively, once all of these covariates have been included. This highlights a significant unexplained component of wellbeing unexplained by young people’s observable characteristics and social support — although we will return to whether more of this difference can be explained by adverse life events during the pandemic in the next section.

The unconditional difference in wellbeing by perceived continuing impact of the pandemic on wellbeing at Wave 2 is lower in models where we have adjusted for Wave 1 wellbeing (0.85 points). However, demographic and social support controls make essentially no difference for this outcome, with the difference remaining 0.81 points once these have been included, with a very similar magnitude to that seen in the fully adjusted model for Wave 1.

We do not find evidence that social support mediates differences in wellbeing by perceived impact of the pandemic (see Fig. 12 in Sect. 8), nor that the differences in wellbeing associated with perceived impact of the pandemic are moderated by young people’s demographic characteristics of socioeconomic background (see Sect. 7).

### Adverse Life Events

**Fig. 5:**
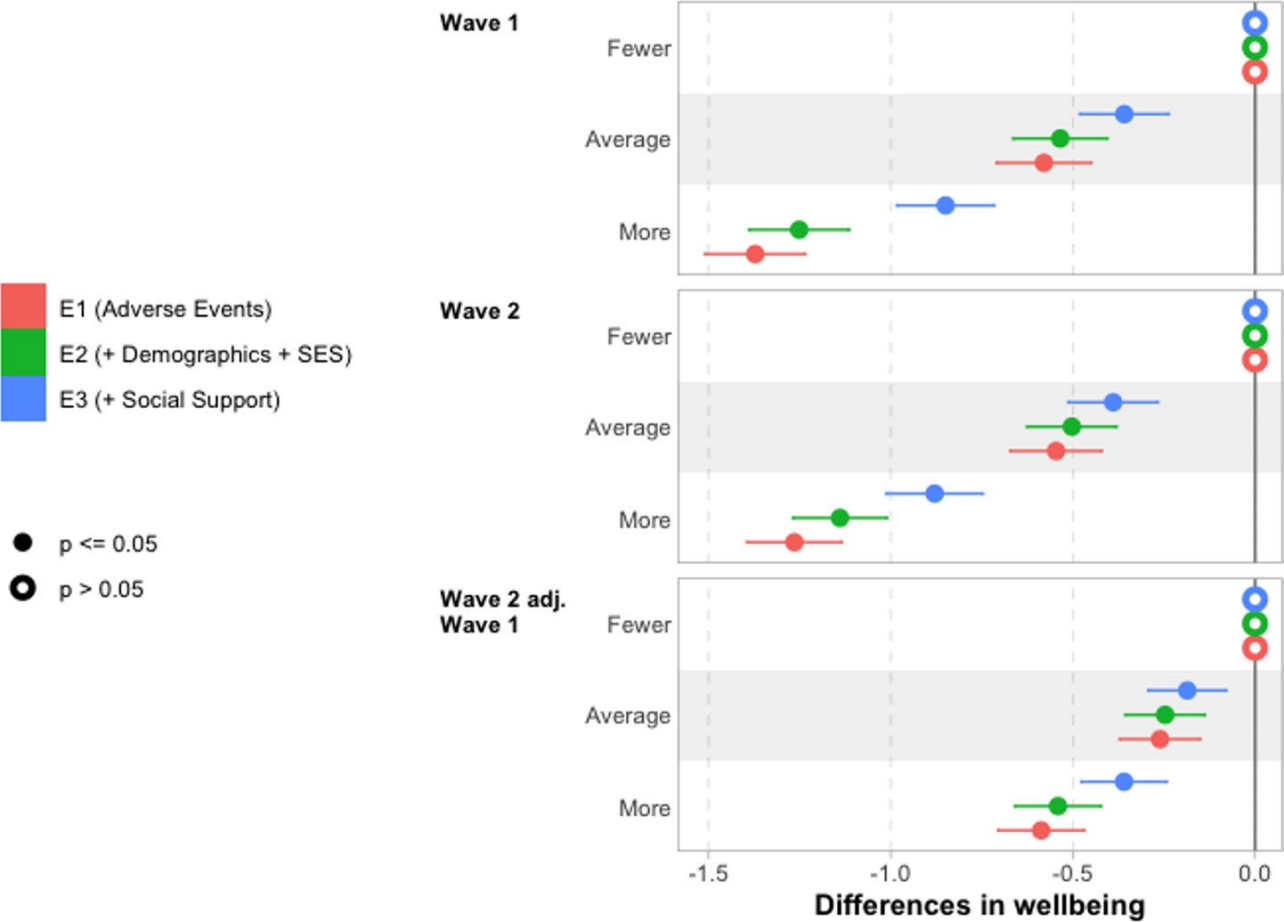
Differences in wellbeing by experience of adverse life events. Notes: Reporting coefficients from underlying regression models reported in Tables 15 and 16, and Table 17.

Next, we turn to the importance of adverse life events for young people’s wellbeing. This is explored through the series of models summarised in Table 6; full results are reported in Tables 15, 16 and 17. in Sect. 7. The core results are plotted in Fig. 5, demonstrating the association unconditionally (E1), adjusting for demographic measures (E2), and adjusting also for social support (E3).

Those who experienced more adverse life events during the pandemic report substantially lower wellbeing, with the unconditional difference between the “fewer” and “more” adverse life events tertile groups being 1.4 points at Wave 1 and 1.3 points at Wave 2. A small part of this is explained by demographics (in E2), while more is explained by social support (in E3), especially for those who experienced the most adverse life events (i.e., the More Adverse Events Tertile Group), bringing the gap between low and high groups to 0.85 points at Wave 1 and 0.88 points at Wave 2.

The patterns are similar but substantially attenuated when considering Wave 2 differences controlling for Wave 1 wellbeing. Nevertheless, there remains a substantial difference (0.36 points) in wellbeing at Wave 2 by adverse events experienced after controlling for Wave 1 wellbeing, demographic characteristics and social support.

**Fig. 6:**
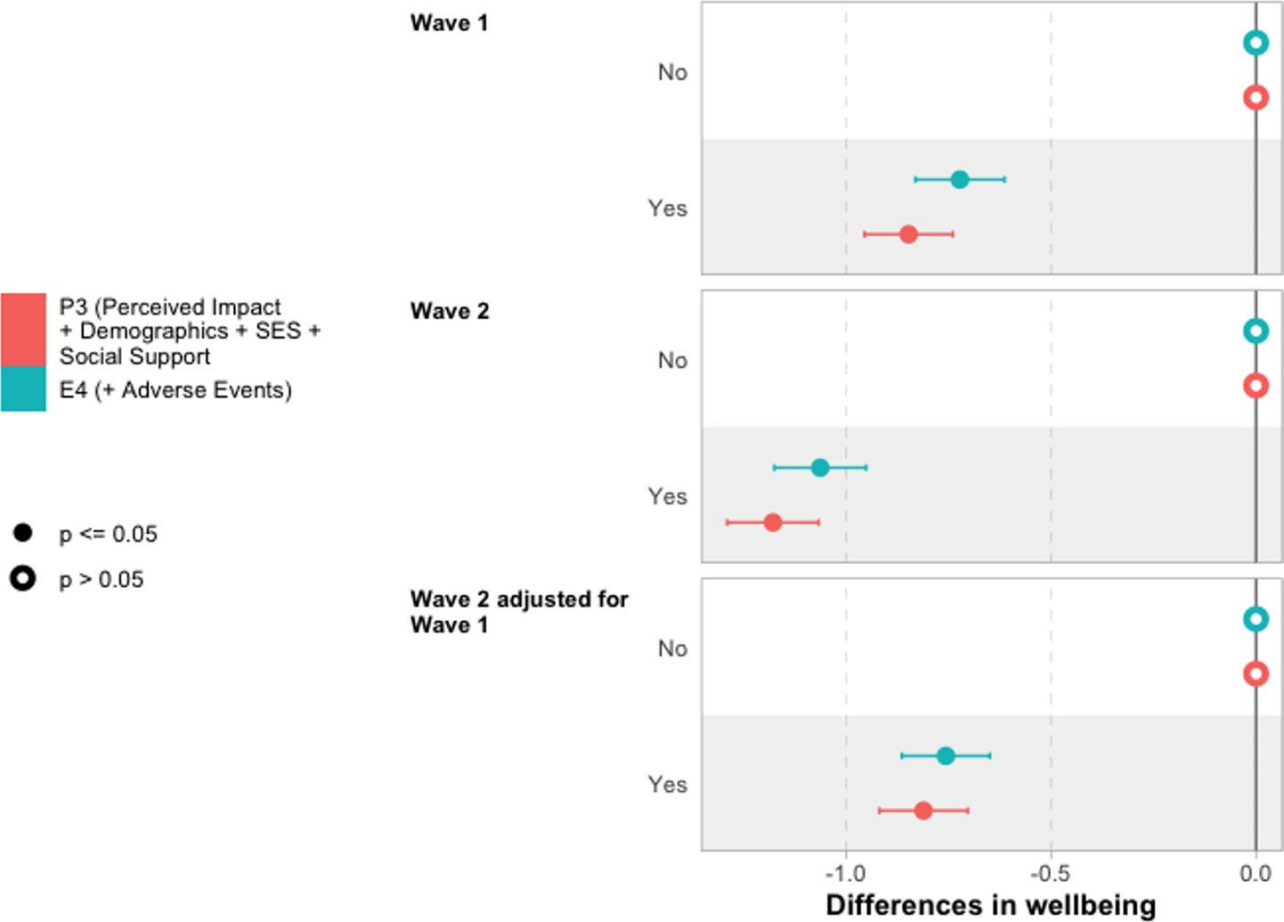
Differences in wellbeing by perceived ongoing negative impact of the pandemic, with and without controlling for adverse life events. Notes: Reporting coefficients from underlying regression models reported in Tables 12 and 13, and Table 14. (for P3), and Tables 15 and 16, and Table 17. (for E4)

**Fig. 9:**
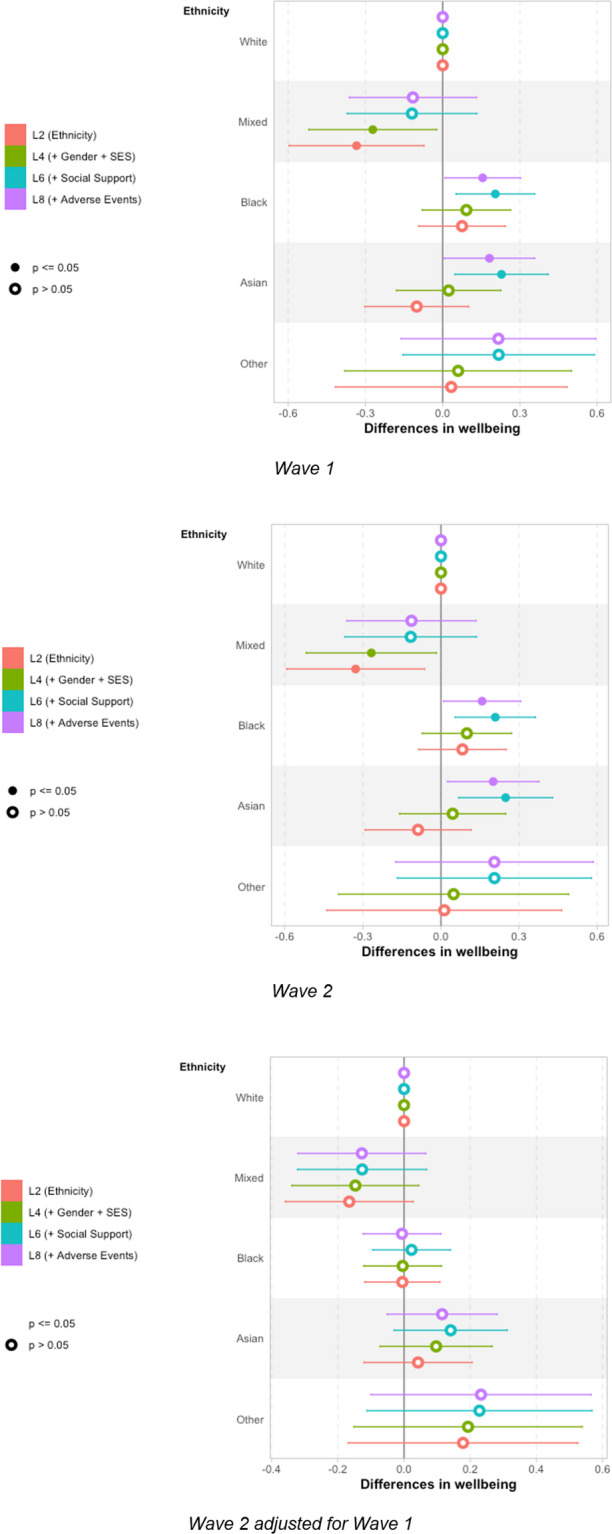
Differences in wellbeing by ethnicity. Notes: Reporting coefficients from underlying regression models reported in Table 9, Table 10, and Table 11

**Fig. 10:**
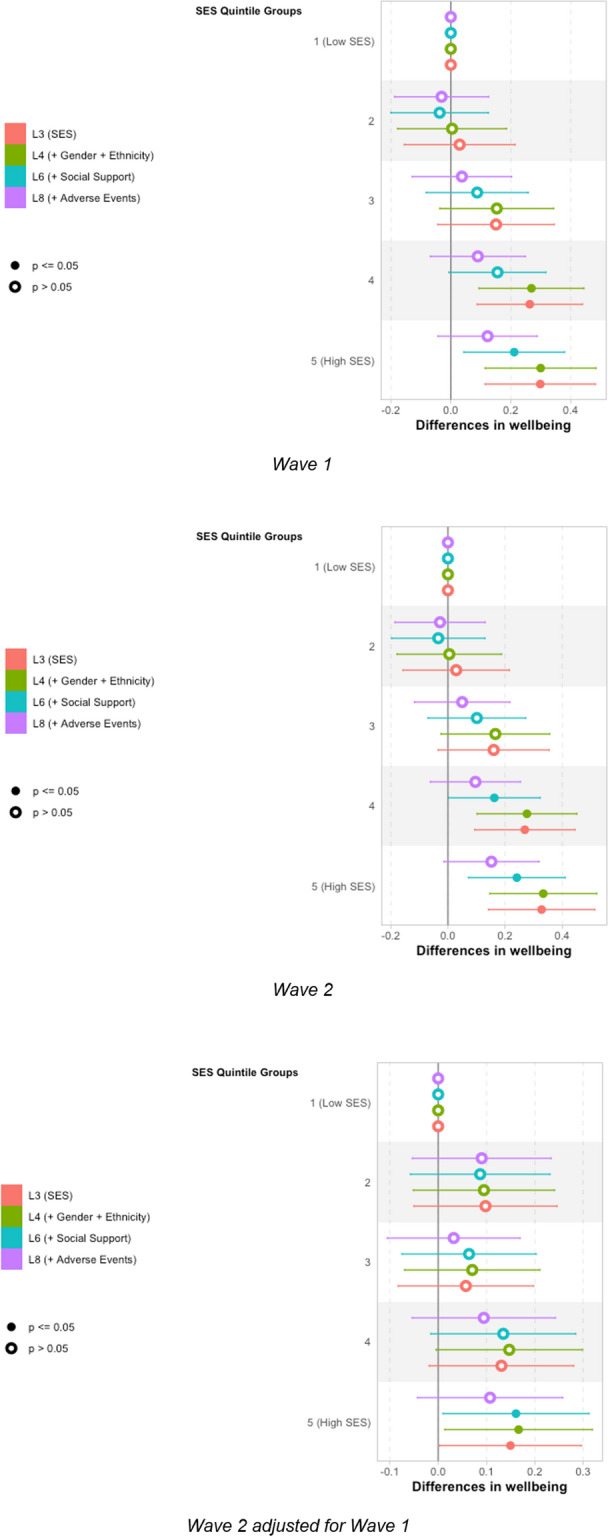
Differences in wellbeing by SES. Notes: Reporting coefficients from underlying regression models reported in Table 9, Table 10, and Table 11

**Fig. 12:**
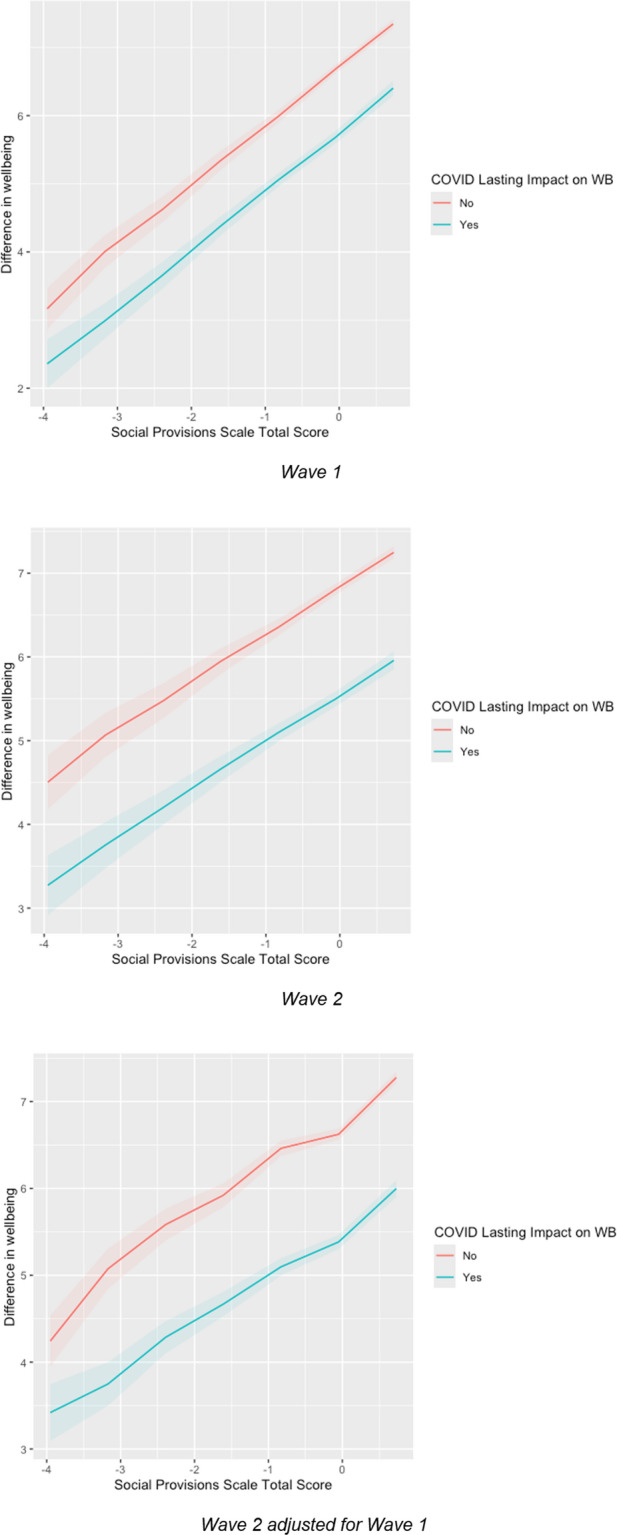
Predicted wellbeing by perceived ongoing impact of pandemic on wellbeing and Social Provisions Scale Total Score. Notes: Predictions derived from underlying regression models, specifically model P4, reported in Table 12, Table 13, and Table 14. Other model characteristics held constant at means or modal values

Building on the models reported in Fig. 5, we also explore whether the association between adverse life events and wellbeing is mediated by the perceived ongoing impact of the pandemic on wellbeing, plotting results in Fig. 6. We find only a small part of perceived ongoing impact of the pandemic on wellbeing is explained by experience of adverse life events during the pandemic.

We also explored whether there was evidence that adverse events matter more for some groups than others, but find little evidence of this. These results are reported in column E5 of Tables 15, 16 and 17. in Sect. 7.

## Discussion, Conclusions and Limitations

This study contributes to existing literature on young people’s wellbeing in England in the aftermath of the COVID-19 pandemic by exploring levels of wellbeing at two time points since the pandemic and the factors associated with these levels. We build on existing work showing that the pandemic has had a negative impact on young people’s wellbeing (e.g., Mansfield et al., 2022), along with evidence of recovery in wellbeing in the latter phases of the pandemic (Henseke et al., 2022; Henseke & Schoon, 2025), although we emphasise that our findings about the state of post-pandemic inequalities do not imply that these have been caused by the pandemic and its restrictions. We have, however, explored the ongoing role of the pandemic through the importance of young people’s own perceptions of its ongoing impact on their wellbeing, as well as the role of adverse life events experienced during the period.

Our results contribute to evidence on gender differences in wellbeing (e.g., Davillas & Jones, 2021). Girls and those who identify as non-binary or in another way report lower wellbeing scores (on a scale from 1 to 10 around 0.5 for girls; around 1.5 for non-binary + young people) than boys. This persists after adjusting for other demographic characteristics, self-reported levels of social support, and experience of adverse life events, contributing to a picture of these inequalities as grounded in a complex set of societally-mediated causes (Guo et al., 2024), which also raise the possibility of variations by gender in the relative importance of different aspects of the social production function (SPF) itself (Steverink et al., 2020), rather than simply that the function’s inputs differ by gender. That said, without analysis of all dimensions of the SPF, this study is not well-placed to provide strong evidence on this point. These inequalities are substantial and relevant to the higher rates of mental health challenges for those in these groups (Yoon et al., 2023). Our findings for non-binary + young people support limited existing evidence (Marquez et al., 2023), but we emphasise that the small sample size of this group in our data means exercising caution in its interpretation.

Our analysis makes innovative use of young people’s own perceptions of the ongoing impact of the pandemic on their mental wellbeing in order to validate and quantify these reports. Our findings illustrate the importance of taking such reports seriously: those who indicate an ongoing negative impact in their lives have substantially lower subjective wellbeing scores — more than 1 point on a 1–10 scale — with similar differences across demographic groups. Moreover, these differences are only partially explained by demographic characteristics, social support, or adverse life events experienced during the pandemic, while there is also little evidence of differential response to a perceived negative impact by socioeconomic background or demographic characteristics. This leaves a substantial difference in wellbeing associated with this perception demonstrating that such perceptions are informative in their own right, analogously to how educational expectations (Anders, 2017) and aspirations (Hart, 2016) can be informative of young people’s educational trajectories over and above other factors. As with that literature, our finding should not be taken to mean such perceptions should be considered causal (Gorard, 2012). While it is probable that there are elements of the SPF that underly these young people’s perceptions, we argue that this does not diminish their informational value and, hence, the importance of taking them seriously (Morgan, 1998). This implies that, nuancing our previous point, there are limits on the extent to which we can target support based on demographic characteristics alone. Self-identification is likely necessary to find those most in need of support, albeit with risks since self-reporting behaviour in a survey likely differs from self-reporting for the purposes of intervention. These findings are similar in spirit to those of Fernandez-Urbano and Samuel (2024), who identify contemporaneous links between how well young people report coping with the pandemic and their subjective wellbeing. We emphasise that the analogy is far from perfect: we are not claiming that a perception of continuing negative impact on wellbeing is the same thing as reporting not coping with the situation, for example. Moreover, unlike Fernandez-Urbano and Samuel (2024), we do not find evidence of differences in response by young people’s socioeconomic status. Nevertheless, these findings align in illustrating the role of such reports in understanding young people’s wellbeing.

Adverse life events experienced during the pandemic are also found to predict lower subjective wellbeing. This is consistent with these undermining aspects of the SPF, such as affection (for events such as arguments within the home) or comfort (in situations of financial distress) (Chesters, 2025), along with previous findings that adverse life events are associated with lower wellbeing (Hombrados-Mendieta et al., 2019; McKnight et al., 2002). However, contrary to our expectations, and others’ findings (Aksoy et al., 2024; Ferreira et al., 2021; Kearns et al., 2015), we did not find evidence that social support mediates or buffers the impact of adverse life events in the context of this study. One potential reason for this is that the source of the social support matters: Lee and Goldstein (2016) find that only support from friends (not family or partners) matters in a study of the stress-buffering role of social support for loneliness. We would expect this to be the source of social support most likely to be cut off by COVID-19 restrictions, although we note that this will not be entirely the case due to the compensatory mechanism of increased use of digital contact between friends in the context of the pandemic (Juvonen et al., 2022). Nevertheless, we contend that this is unlikely entirely successfully to replicate the benefits of in-person contact (Flannery et al., 2021; Long et al., 2022). More methodologically, with hindsight we note that, while our measures of social support are contemporaneous with our wellbeing measures, they are not contemporaneous with the timing of the adverse events themselves, which may mean they are not providing an accurate depiction of perceived social support during pandemic disruption.

This study benefits from a large, representative, longitudinal dataset, with direct reports from both young people and parents to improve the quality of data collected. Nevertheless, we are mindful of the limitations of this study, most particularly that our data lacks pre-pandemic baseline measures of wellbeing, which would substantially increase our ability to understand the longer-term dynamics of the changes (or lack thereof) in wellbeing that we have observed. Furthermore, our data is drawn from a single cohort of young people in England, whose final years in compulsory education were especially disrupted by the impacts of the pandemic, which is important context in any attempt to generalise our findings to other populations. As noted above, we also emphasise the small sample size of our non-binary + group, which limits the robustness of findings for this group.

Our findings indicate continuing challenges of inequalities in young people’s wellbeing and, hence, the importance of ongoing targeted support to overcome these. We reiterate, however, that we do not link these inequalities particularly with lingering effects of the pandemic since we did not find evidence of differences in the association between perceived continuing impact of the pandemic on wellbeing and young people’s socioeconomic or demographic characteristics. However, in some ways inequalities not linked to the pandemic are of greater concern, given little reason to expect them to subside without intervention. As such, the large differences in wellbeing associated with identifying as non-binary or in another way may suggest a particular need for support among this group, although we reiterate the small sample sizes involved in findings for those identifying as neither male nor female, making further evidence for this group especially important. The practicalities of providing support at scale are now much harder for our specific cohort, since many have now left education entirely. Nevertheless, the issues discussed will apply similarly to those still working their way through the education system who could be reached through schools and colleges. As well as the negative implications for the life experiences of these young people, ignoring this issue has potential implications for national economic performance (Deaton, 2008), including via increased risk of mental health challenges (Lombardo et al., 2018).

## Figures and Tables

**Fig. 7:**
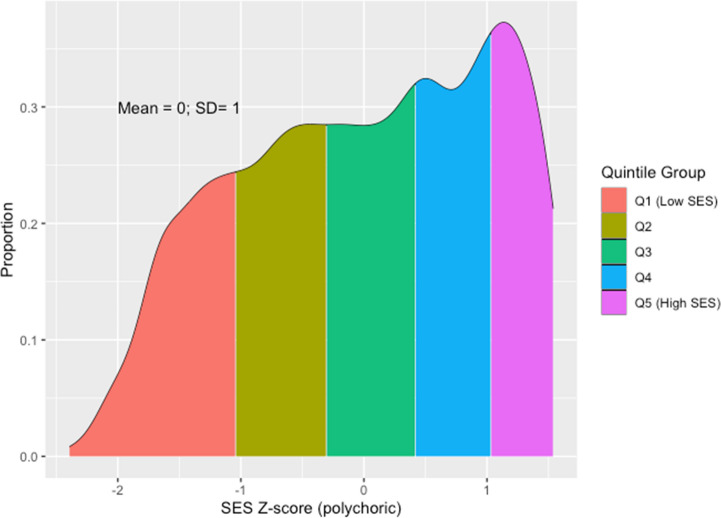
Distribution of SES summary measure, colour-coded by quintile group. Notes: SES measure based on polychoric principal component analysis of parental education, housing tenure and IDACI decile group. Density plot weighted for survey design and non-response

**Fig. 8:**
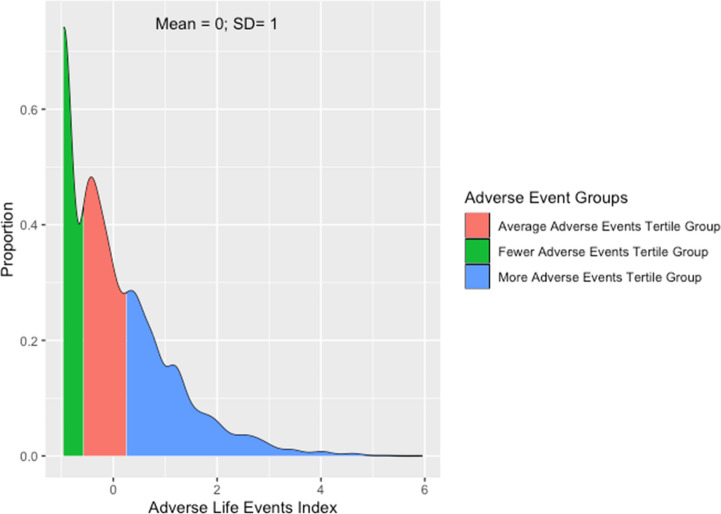
Distribution of Adverse Events Index, colour-coded by tertile group. **Notes**: Adverse events index based on polychoric principal component analysis of measured adverse life events. The index is standardised to have mean 0 and standard deviation 1 in the analysis sample. Weighted for survey design and non-response

**Fig. 11:**
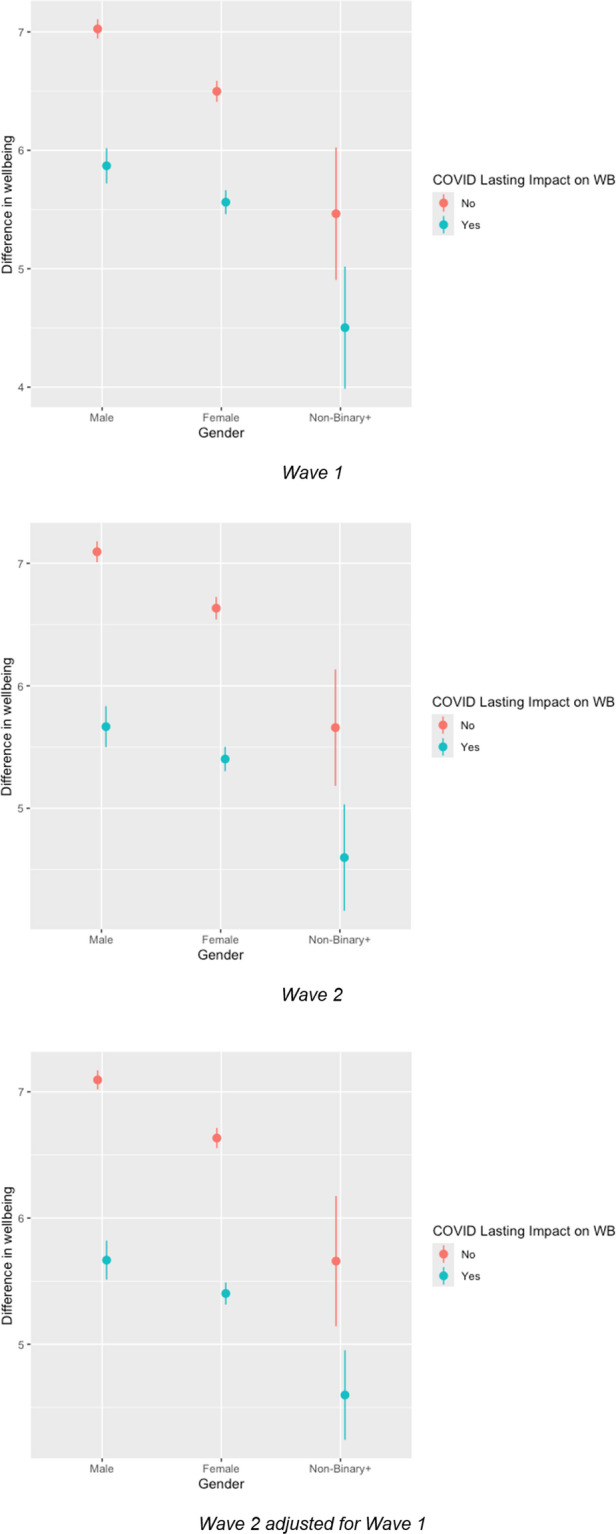
Predicted wellbeing by perceived ongoing impact of pandemic on wellbeing and gender. Notes: Predictions derived from underlying regression models reported in Table 12, Table 13, and Table 14

## Appendix: Construction of measures

### Construction of SES measure

To allow exploration of differences in wellbeing by socioeconomic status (SES) in a simple way, we create a combined index of SES across our measures of parental education, housing tenure and home neighbourhood deprivation. Specifically, given the categorical nature of these variables, we estimate a polychoric correlation matrix of these measures and use principal component analysis (Revelle, 2025) to extract a single component that explains maximum shared variance. Our extracted principal component score explains 65% of the overall variance of our SES measures. We standardise the measure’s distribution to have mean 0 and standard deviation 1 in our analysis sample, plot its distribution in Fig. 7, and use it to split our sample into five quintile groups of equal size (accounting for sample weighting).

We demonstrate that this measure captures the underlying SES measures on which it is based in Table 7 by reporting the average levels of parental education, housing tenure and IDACI quintile group across the five quintile groups of the constructed SES measure.

**Table 7:** Distribution of underlying socioeconomic characteristics by SES quintile group (SES quintile group based on polychoric principal component analysis of parental education, housing tenure and IDACI decile group)

Characteristic	1 (Low SES) N = 1,602	2 N = 1,598	3 N = 1,608	4 N = 1,665	5 (High SES) N = 1,519
Parental Education					
Graduate	16	41	63	69	89
Below Graduate	54	52	33	30	11
No Quals	27	6.3	3.3	1.4	0
Unknown	3.1	0.8	0.2	0	0
Housing Tenure					
Own House	10	49	75	90	100
Other	90	51	25	9.5	0
Unknown	0	0	0	0	0
IDACI Quintile Group					
1 (High Deprivation)	76	31	1.8	0	0
2	23	43	29	<0.1	0
3	1.1	23	47	22	0
4	<0.1	3.6	18	60	17
5 (Low Deprivation)	0	0.2	3.3	19	83

Reporting column percentages within each variable. All estimates are weighted for survey design and non-response

### Construction of Adverse Life Events Measure

In Wave 1, COSMO asked participants whether they had experienced each of the following life events since the onset of the pandemic in March 2020:

1. A parent/guardian or carer lost their job or business
2. My family could not afford to buy enough food, or had to use a food bank
3. My family could not afford to pay their bills/rent/mortgage
4. I was seriously ill in hospital
5. A close family member or friend is or was seriously ill in hospital
6. A close family member or friend died
7. Increase in number of arguments with parents/guardians
8. Increase in number of arguments between parents/guardians
9. Moving to a new home
10. Parents/guardians separated or divorced

The question is worded to capture events whether or not they are directly attributable to the pandemic, its restrictions and disruptions, but it is reasonable to believe many were caused or exacerbated by the circumstances of the pandemic. Participants were then asked whether they had experienced these events over the past twelve months (i.e., for most participants a year since they responded to the Wave 1 survey) in Wave 2.

29% of pupils experience no events at all, while 26% experience three or more events. We report the proportion of young people experiencing each of the ten specific adverse life events in the final column of Table 8.

**Table 8:** Adverse life events experienced by Adverse Events Index group

Variable, *N* = 7723	Fewer (36%)^1^	Average (30%)^1^	More (33%)^1^	Overall (100%)^1^
Parent lost job	0	13	23	12
Couldn’t afford food	0	2.8	23	8.4
Couldn’t afford bills	0	4.6	28	11
Seriously ill	0	2.6	7.0	3.1
Close family member seriously ill	0	45	54	32
Close family member died	19	28	51	33
More arguments with parents	0	28	72	32
More arguments between parents	0	7.8	60	22
Moved home	0	6.6	16	7.3
Parents separated	0	1.5	10	3.8
Number of events (grouped)				
0	81	0	0	29
1	19	60	0	25
2	0	40	23	20
3+	0	0	77	26
Number of events (mean)	0.19	1.40	3.44	1.64

1%; Mean

All estimates are weighted and account for the complex survey design

The substantial differences in prevalence of the events means that using a simple count of events experienced would inappropriately impose the same importance, or severity, for all the events. Instead, we allow these to differ, such that lower probability/higher impact events are given more weight by creating a composite index of adverse life events using polychoric principal component analysis (PCA) of the ten adverse life events.

The first principal component explains 32% of the variance. We standardise this index (mean 0; standard deviation 1) in our analysis sample, plot the distribution in Fig. 8, and split it into three groups based on the tertiles of the index (accounting for sample weighting). We label these groups as “Fewer Adverse Events Tertile Group”, “Average Adverse Events Tertile Group” and “More Adverse Events Tertile Group” to reflect the relative impact of adverse events experienced in each.

We report the prevalence of each of the adverse life events by the three groups in Table 8. This demonstrates that these groups are capturing different levels of exposure to adverse life events, while reflecting the differential prevalence of the events. Students in the “Fewer Adverse Events Tertile Group” are unlikely to have experienced any of the events, with the exception of a close family member dying. In contrast, students in the “More Adverse Events Tertile Group” are likely to have experienced multiple events.

## Appendix: Full Regression Tables

### Demographic Differences in Wellbeing

**Table 9:** Differences in wellbeing at wave 1

	L1		L2		L3		L4		L5		L6		L7		L8	
Characteristic	**Beta** ^*1*^	**SE**	**Beta** ^*1*^	**SE**	**Beta** ^*1*^	**SE**	**Beta** ^*1*^	**SE**	**Beta** ^*1*^	**SE**	**Beta** ^*1*^	**SE**	**Beta** ^*1*^	**SE**	**Beta** ^*1*^	**SE**
(Intercept)	6.7***	0.062	6.4***	0.057	6.2***	0.076	6.6***	0.087	6.5***	0.117	6.6***	0.081	6.6***	0.081	6.6***	0.079
Gender																
Male	—	—					—	—	—	—	—	—	—	—	—	—
Female	−0.63***	0.057					−0.63***	0.056	−0.53***	0.123	−0.60***	0.051	−0.60***	0.051	−0.50***	0.051
Non-Binary+	−1.9***	0.216					−1.9***	0.218	−1.7***	0.387	−1.4***	0.208	−1.4***	0.230	−1.2***	0.202
Ethnicity																
White			—	—			—	—	—	—	—	—	—	—	—	—
Mixed			−0.33*	0.133			−0.27*	0.127	−0.34	0.278	−0.12	0.128	−0.14	0.124	−0.12	0.126
Black			0.08	0.086			0.09	0.087	0.20	0.191	0.20**	0.078	0.20*	0.077	0.16*	0.075
Asian			−0.10	0.103			0.02	0.103	0.13	0.222	0.23*	0.092	0.22*	0.091	0.18*	0.089
Other			0.03	0.229			0.06	0.224	0.16	0.513	0.22	0.189	0.22	0.184	0.22	0.193
SES Quintile Groups																
1 (Low SES)					—	—	—	—	—	—	—	—	—	—	—	—
2					0.03	0.094	0.00	0.093	0.05	0.146	−0.04	0.083	−0.03	0.081	−0.03	0.080
3					0.15	0.099	0.15	0.097	0.23	0.163	0.09	0.087	0.09	0.086	0.04	0.085
4					0.26**	0.090	0.27**	0.089	0.33*	0.140	0.16	0.082	0.16	0.082	0.09	0.081
5 (High SES)					0.30**	0.094	0.30**	0.094	0.36*	0.142	0.21*	0.086	0.21*	0.085	0.12	0.084
SES Quintile Groups * Gender																
2 * Female									−0.09	0.175						
3 * Female									−0.24	0.185						
4 * Female									−0.05	0.170						
5 (High SES) * Female									−0.01	0.172						
2 * Non-Binary+									−0.23	0.553						
3 * Non-Binary+									0.46	0.697						
4 * Non-Binary+									0.23	0.588						
5 (High SES) * Non-Binary+									−1.3*	0.587						
SES Quintile Groups * Ethnicity																
2 * Mixed									0.29	0.387						
3 * Mixed									0.26	0.363						
4 * Mixed									−0.25	0.333						
5 (High SES) * Mixed									0.11	0.332						
2 * Black									−0.02	0.246						
3 * Black									−0.05	0.239						
4 * Black									−0.23	0.287						
5 (High SES) * Black									−0.35	0.303						
2 * Asian									−0.11	0.220						
3 * Asian									0.07	0.258						
4 * Asian									0.04	0.387						
5 (High SES) * Asian									−0.66	0.935						
2 * Other									−0.06	0.551						
3 * Other									0.78	0.672						
4 * Other									−0.15	0.664						
5 (High SES) * Other									−0.08	0.949						
Gender * Ethnicity																
Female * Mixed									0.01	0.249						
Non-Binary+ * Mixed									−0.22	0.617						
Female * Black									−0.09	0.161						
Non-Binary+ * Black									0.43	0.678						
Female * Asian									−0.09	0.220						
Non-Binary+ * Asian									−0.92	1.03						
Female * Other									−0.36	0.454						
Non-Binary+ * Other									1.4*	0.613						
Social Provisions Scale											0.90***	0.028	0.89***	0.075	0.83***	0.029
Gender * Social Provisions Scale																
Female * Social Provisions Scale													0.02	0.054		
Non-Binary+ * Social Provisions Scale													−0.07	0.157		
Ethnicity * Social Provisions Scale																
Mixed * Social Provisions Scale													−0.17	0.109		
Black * Social Provisions Scale													−0.12	0.074		
Asian * Social Provisions Scale													−0.09	0.083		
Other * Social Provisions Scale													0.03	0.194		
SES Quintile Groups * Social Provisions Scale																
2 * Social Provisions Scale													0.08	0.081		
3 * Social Provisions Scale													0.06	0.083		
4 * Social Provisions Scale													−0.02	0.089		
5 (High SES) * Social Provisions Scale													0.03	0.086		
Adverse Event Index															−0.35***	0.028
W1 Month of Interview																
Sep 2021	—	—	—	—	—	—	—	—	—	—	—	—	—	—	—	—
Oct 2021	0.10	0.067	0.14*	0.067	0.12	0.067	0.09	0.068	0.10	0.068	0.03	0.061	0.03	0.061	0.03	0.060
Nov 2021	0.41*	0.193	0.42*	0.185	0.37*	0.184	0.36	0.188	0.37*	0.183	0.38*	0.169	0.39*	0.169	0.38*	0.165
Dec 2021	0.32*	0.135	0.30*	0.135	0.30*	0.135	0.29*	0.132	0.31*	0.135	0.14	0.116	0.14	0.116	0.13	0.113
Jan 2022	0.49	0.250	0.50	0.260	0.51*	0.257	0.48	0.246	0.47*	0.241	0.47	0.260	0.47	0.262	0.43	0.256
Feb 2022	−0.33	0.234	−0.21	0.251	−0.23	0.246	−0.37	0.233	−0.36	0.229	−0.50*	0.243	−0.49*	0.243	−0.49*	0.246
Mar 2022	−0.12	0.093	−0.10	0.095	−0.10	0.096	−0.12	0.093	−0.12	0.093	−0.14	0.084	−0.14	0.084	−0.13	0.084
Apr 2022	−0.04	0.102	0.00	0.105	−0.01	0.106	−0.06	0.103	−0.05	0.102	−0.08	0.096	−0.08	0.096	−0.10	0.093
N	7,723		7,723		7,723		7,723		7,723		7,723		7,723		7,723	
Residual DoF	757		755		755		749		717		748		738		747	

^1^*p<0.05; **p<0.01; ***p<0.001

Abbreviations:*CI* Confidence Interval,*SE* Standard Error

All estimates are weighted and inference accounts for the complex survey design

**Table 10:** Differences in wellbeing at wave 2

	L1		L2		L3		L4		L5		L6		L7		L8	
Characteristic	**Beta** ^*1*^	**SE**	**Beta** ^*1*^	**SE**	**Beta** ^*1*^	**SE**	**Beta** ^*1*^	**SE**	**Beta** ^*1*^	**SE**	**Beta** ^*1*^	**SE**	**Beta** ^*1*^	**SE**	**Beta** ^*1*^	**SE**
(Intercept)	6.8***	0.050	6.4***	0.043	6.3***	0.069	6.6***	0.081	6.6***	0.113	6.7***	0.075	6.6***	0.074	6.7***	0.073
Gender																
Male	—	—					—	—	—	—	—	—	—	—	—	—
Female	−0.63***	0.057					−0.63***	0.057	−0.55***	0.123	−0.60***	0.051	−0.60***	0.051	−0.51***	0.051
Non-Binary+	−1.9***	0.212					−1.8***	0.214	−1.7***	0.383	−1.4***	0.204	−1.4***	0.224	−1.2***	0.199
Ethnicity																
White			—	—			—	—	—	—	—	—	—	—	—	—
Mixed			−0.33*	0.135			−0.27*	0.127	−0.32	0.279	−0.12	0.129	−0.14	0.125	−0.11	0.126
Black			0.08	0.086			0.10	0.088	0.22	0.192	0.21**	0.079	0.20*	0.078	0.16*	0.075
Asian			−0.09	0.104			0.04	0.104	0.14	0.221	0.25**	0.092	0.24**	0.091	0.20*	0.089
Other			0.01	0.230			0.05	0.225	0.15	0.515	0.21	0.190	0.21	0.185	0.21	0.194
SES Quintile Groups																
1 (Low SES)					—	—	—	—	—	—	—	—	—	—	—	—
2					0.03	0.095	0.00	0.093	0.05	0.147	−0.03	0.083	−0.03	0.082	−0.03	0.080
3					0.16	0.099	0.17	0.097	0.25	0.163	0.10	0.087	0.10	0.087	0.05	0.085
4					0.27**	0.089	0.28**	0.089	0.32*	0.140	0.16*	0.082	0.16*	0.082	0.10	0.080
5 (High SES)					0.33***	0.095	0.33***	0.095	0.39**	0.141	0.24**	0.086	0.24**	0.086	0.15	0.085
SES Quintile Groups * Gender																
2 * Female									−0.08	0.175						
3 * Female									−0.22	0.186						
4 * Female									−0.03	0.170						
5 (High SES) * Female									0.01	0.175						
2 * Non-Binary+									−0.26	0.545						
3 * Non-Binary+									0.46	0.690						
4 * Non-Binary+									0.24	0.584						
5 (High SES) * Non-Binary+									−1.3*	0.576						
SES Quintile Groups * Ethnicity																
2 * Mixed									0.27	0.388						
3 * Mixed									0.22	0.365						
4 * Mixed									−0.24	0.337						
5 (High SES) * Mixed									0.06	0.335						
2 * Black									−0.04	0.243						
3 * Black									−0.10	0.237						
4 * Black									−0.25	0.289						
5 (High SES) * Black									−0.35	0.308						
2 * Asian									−0.13	0.220						
3 * Asian									0.05	0.258						
4 * Asian									0.05	0.395						
5 (High SES) * Asian									−0.54	0.996						
2 * Other									−0.05	0.555						
3 * Other									0.75	0.675						
4 * Other									−0.14	0.660						
5 (High SES) * Other									−0.08	0.981						
Gender * Ethnicity																
Female * Mixed									0.01	0.252						
Non-Binary+ * Mixed									−0.19	0.603						
Female * Black									−0.06	0.161						
Non-Binary+ * Black									0.41	0.694						
Female * Asian									−0.06	0.221						
Non-Binary+ * Asian									−0.91	1.08						
Female * Other									−0.37	0.454						
Non-Binary+ * Other									1.4*	0.663						
Social Provisions Scale											0.90***	0.028	0.88***	0.075	0.83***	0.029
Gender * Social Provisions Scale																
Female * Social Provisions Scale													0.03	0.055		
Non-Binary+ * Social Provisions Scale													−0.07	0.154		
Ethnicity * Social Provisions Scale																
Mixed * Social Provisions Scale													−0.18	0.108		
Black * Social Provisions Scale													−0.11	0.073		
Asian * Social Provisions Scale													−0.08	0.083		
Other * Social Provisions Scale													0.03	0.195		
SES Quintile Groups * Social Provisions Scale																
2 * Social Provisions Scale													0.08	0.082		
3 * Social Provisions Scale													0.07	0.083		
4 * Social Provisions Scale													−0.01	0.090		
5 (High SES) * Social Provisions Scale													0.03	0.088		
Adverse Event Index															−0.35***	0.028
W2 Month of Survey																
October 2022	—	—	—	—	—	—	—	—	—	—	—	—	—	—	—	—
November 2022	−0.07	0.059	−0.03	0.061	−0.04	0.061	−0.09	0.059	−0.08	0.059	−0.09	0.053	−0.09	0.053	−0.11*	0.052
December 2022	−0.13	0.133	−0.09	0.141	−0.10	0.140	−0.15	0.133	−0.14	0.132	−0.16	0.121	−0.16	0.121	−0.16	0.118
January 2023	−0.31	0.222	−0.21	0.222	−0.20	0.226	−0.31	0.227	−0.31	0.225	−0.38	0.201	−0.37	0.200	−0.39*	0.188
February 2023	0.62**	0.211	0.65**	0.199	0.66**	0.201	0.60**	0.214	0.60**	0.210	0.48*	0.192	0.48*	0.191	0.43*	0.185
March 2023	−0.16	0.212	−0.09	0.213	−0.07	0.211	−0.16	0.209	−0.17	0.208	−0.09	0.203	−0.10	0.203	−0.09	0.204
April 2023	−0.04	0.226	−0.01	0.230	−0.05	0.227	−0.07	0.223	−0.06	0.224	0.12	0.179	0.12	0.178	0.11	0.177
N	7,723		7,723		7,723		7,723		7,723		7,723		7,723		7,723	
Residual DoF	758		756		756		750		718		749		739		748	

^1^*p<0.05; **p<0.01; ***p<0.001

Abbreviations: *CI * Confidence Interval, *SE* Standard Error

All estimates are weighted and inference accounts for the complex survey design

**Table 11:** Differences in wellbeing at wave 2 (conditional on wave 1 wellbeing)

	L1		L2		L3		L4		L5		L6		L7		L8	
Characteristic	**Beta** ^*1*^	**SE**	**Beta** ^*1*^	**SE**	**Beta** ^*1*^	**SE**	**Beta** ^*1*^	**SE**	**Beta** ^*1*^	**SE**	**Beta** ^*1*^	**SE**	**Beta** ^*1*^	**SE**	**Beta** ^*1*^	**SE**
(Intercept)	3.3***	0.118	3.0***	0.106	2.9***	0.112	3.2***	0.126	3.1***	0.140	3.5***	0.138	3.5***	0.138	3.6***	0.138
Gender																
Male	—	—					—	—	—	—	—	—	—	—	—	—
Female	−0.28***	0.046					−0.29***	0.046	−0.20	0.114	−0.31***	0.046	−0.31***	0.047	−0.26***	0.046
Non-Binary+	−0.77***	0.169					−0.75***	0.169	−1.1**	0.349	−0.74***	0.165	−0.70***	0.173	−0.66***	0.162
Wave 1 Wellbeing	0.51***	0.015	0.53***	0.014	0.53***	0.014	0.51***	0.015	0.51***	0.015	0.47***	0.017	0.47***	0.017	0.45***	0.017
Ethnicity																
White			—	—			—	—	—	—	—	—	—	—	—	—
Mixed			−0.17	0.098			−0.15	0.098	0.01	0.242	−0.13	0.099	−0.12	0.100	−0.13	0.098
Black			−0.01	0.058			0.00	0.060	0.25	0.132	0.02	0.060	0.02	0.059	−0.01	0.060
Asian			0.04	0.083			0.10	0.086	0.25	0.158	0.14	0.087	0.10	0.086	0.11	0.084
Other			0.18	0.177			0.19	0.176	0.30	0.386	0.23	0.173	0.25	0.166	0.23	0.169
SES Quintile Groups																
1 (Low SES)					—	—	—	—	—	—	—	—	—	—	—	—
2					0.10	0.075	0.09	0.074	0.14	0.124	0.09	0.074	0.08	0.073	0.09	0.073
3					0.06	0.071	0.07	0.071	0.13	0.123	0.06	0.071	0.06	0.071	0.03	0.070
4					0.13	0.076	0.15	0.077	0.22	0.125	0.13	0.076	0.13	0.077	0.09	0.075
5 (High SES)					0.15*	0.075	0.17*	0.078	0.21	0.114	0.16*	0.077	0.16*	0.077	0.11	0.076
SES Quintile Groups * Gender																
2 * Female									−0.07	0.150						
3 * Female									−0.03	0.148						
4 * Female									−0.07	0.154						
5 (High SES) * Female									−0.05	0.144						
2 * Non-Binary+									−0.06	0.403						
3 * Non-Binary+									0.68	0.424						
4 * Non-Binary+									0.09	0.522						
5 (High SES) * Non-Binary+									0.54	0.563						
SES Quintile Groups * Ethnicity																
2 * Mixed									−0.24	0.335						
3 * Mixed									−0.27	0.244						
4 * Mixed									−0.11	0.287						
5 (High SES) * Mixed									0.03	0.292						
2 * Black									−0.13	0.165						
3 * Black									−0.39*	0.178						
4 * Black									−0.20	0.200						
5 (High SES) * Black									−0.42	0.216						
2 * Asian									−0.09	0.208						
3 * Asian									−0.13	0.234						
4 * Asian									0.17	0.213						
5 (High SES) * Asian									0.77	0.775						
2 * Other									0.85	0.437						
3 * Other									0.30	0.414						
4 * Other									−0.93*	0.448						
5 (High SES) * Other									−0.77	0.700						
Gender * Ethnicity																
Female * Mixed									−0.13	0.212						
Non-Binary+ * Mixed									0.54	0.478						
Female * Black									−0.16	0.114						
Non-Binary+ * Black									−0.14	0.654						
Female * Asian									−0.27	0.168						
Non-Binary+ * Asian									2.0**	0.665						
Female * Other									−0.35	0.332						
Non-Binary+ * Other									0.42	0.433						
Social Provisions Scale											0.19***	0.029	0.18**	0.067	0.16***	0.029
Gender * Social Provisions Scale																
Female * Social Provisions Scale													0.03	0.055		
Non-Binary+ * Social Provisions Scale													0.09	0.126		
Ethnicity * Social Provisions Scale																
Mixed * Social Provisions Scale													0.03	0.088		
Black * Social Provisions Scale													0.00	0.068		
Asian * Social Provisions Scale													−0.21**	0.078		
Other * Social Provisions Scale													0.11	0.174		
SES Quintile Groups * Social Provisions Scale																
2 * Social Provisions Scale													−0.06	0.073		
3 * Social Provisions Scale													−0.01	0.076		
4 * Social Provisions Scale													0.03	0.084		
5 (High SES) * Social Provisions Scale													0.01	0.079		
Adverse Event Index															−0.23***	0.024
W2 Month of Survey																
October 2022	—	—	—	—	—	—	—	—	—	—	—	—	—	—	—	—
November 2022	−0.03	0.049	−0.02	0.050	−0.02	0.050	−0.04	0.050	−0.05	0.050	−0.05	0.050	−0.04	0.050	−0.06	0.050
December 2022	0.24*	0.103	0.26*	0.105	0.26*	0.104	0.23*	0.103	0.23*	0.102	0.23*	0.103	0.23*	0.102	0.22*	0.100
January 2023	0.42	0.255	0.46	0.257	0.47	0.256	0.42	0.253	0.40	0.253	0.39	0.256	0.40	0.256	0.37	0.263
February 2023	0.31	0.208	0.32	0.204	0.33	0.206	0.31	0.210	0.29	0.210	0.31	0.203	0.31	0.201	0.29	0.205
March 2023	0.39**	0.139	0.43**	0.138	0.43**	0.139	0.39**	0.141	0.37**	0.140	0.40**	0.142	0.40**	0.141	0.39**	0.146
April 2023	0.16	0.183	0.16	0.185	0.16	0.185	0.14	0.184	0.16	0.183	0.18	0.180	0.18	0.180	0.18	0.183
N	7,723		7,723		7,723		7,723		7,723		7,723		7,723		7,723	
Residual DoF	757		755		755		749		717		748		738		747	

^1^*p<0.05; **p<0.01; ***p<0.001

Abbreviations: *CI* Confidence Interval, *SE* Standard Error

All estimates are weighted and inference accounts for the complex survey design

### Perceived Continuing Impact of the Pandemic On Wellbeing

**Table 12:** Differences in wellbeing at wave 1 by perceived continuing impact of pandemic on wellbeing

	P1		P2		P3		P4	
Characteristic	**Beta** ^*1*^	**SE**	**Beta** ^*1*^	**SE**	**Beta** ^*1*^	**SE**	**Beta**^*1*^>	**SE**
(Intercept)	6.7***	0.057	6.9***	0.103	6.9***	0.092	6.9***	0.106
Negative continuing impact of pandemic on mental wellbeing								
No	—	—	—	—	—	—	—	—
Yes	−1.1***	0.062	−1.0***	0.062	−0.85***	0.055	−1.0***	0.181
Gender								
Male			—	—	—	—	—	—
Female			−0.46***	0.057	−0.47***	0.051	−0.55***	0.061
Non-Binary+			−1.5***	0.213	−1.1***	0.205	−1.2***	0.288
Ethnicity								
White			—	—	—	—	—	—
Mixed			−0.28*	0.123	−0.13	0.125	−0.13	0.151
Black			0.04	0.082	0.15*	0.076	0.19*	0.089
Asian			0.00	0.102	0.19*	0.092	0.12	0.111
Other			0.09	0.219	0.22	0.187	0.22	0.227
Parental Education								
Graduate			—	—	—	—	—	—
Below Graduate			−0.07	0.066	−0.05	0.059	−0.09	0.071
No Quals			−0.22	0.124	−0.09	0.115	−0.10	0.144
Unknown			−0.09	0.303	0.14	0.331	0.18	0.401
Housing Tenure								
Own House			—	—	—	—	—	—
Other			−0.10	0.066	−0.06	0.062	0.01	0.076
IDACI Quintile Group								
1 (High Deprivation)			—	—	—	—	—	—
2			0.14	0.093	0.09	0.085	0.08	0.103
3			0.07	0.098	0.03	0.089	0.01	0.108
4			0.22*	0.097	0.19*	0.088	0.08	0.104
5 (Low Deprivation)			0.27**	0.103	0.21*	0.093	0.24*	0.112
Social Provisions Scale					0.86***	0.028	0.86***	0.035
Negative continuing impact of pandemic on mental wellbeing * Gender								
Yes * Female							0.27*	0.109
Yes * Non-Binary+							0.32	0.387
Negative continuing impact of pandemic on mental wellbeing * Ethnicity								
Yes * Mixed							0.00	0.216
Yes * Black							−0.14	0.162
Yes * Asian							0.23	0.184
Yes * Other							−0.05	0.416
Negative continuing impact of pandemic on mental wellbeing * Parental Education								
Yes * Below Graduate							0.11	0.124
Yes * No Quals							0.01	0.227
Yes * Unknown							−0.23	0.589
Negative continuing impact of pandemic on mental wellbeing * Housing Tenure								
Yes * Other							−0.22	0.126
Negative continuing impact of pandemic on mental wellbeing * IDACI Quintile Group								
Yes * 2							0.06	0.169
Yes * 3							0.08	0.189
Yes * 4							0.35	0.183
Yes * 5 (Low Deprivation)							−0.08	0.199
Negative continuing impact of pandemic on mental wellbeing * Social Provisions Scale								
Yes * Social Provisions Scale							−0.02	0.052
W1 Month of Interview								
Sep 2021	—	—	—	—	—	—	—	—
Oct 2021	0.12	0.067	0.07	0.067	0.02	0.060	0.02	0.060
Nov 2021	0.37*	0.185	0.29	0.184	0.33	0.169	0.33	0.167
Dec 2021	0.25	0.132	0.23	0.132	0.10	0.114	0.10	0.112
Jan 2022	0.48	0.254	0.45	0.234	0.45	0.256	0.43	0.258
Feb 2022	−0.39	0.235	−0.52*	0.225	−0.62**	0.233	−0.61*	0.236
Mar 2022	−0.08	0.093	−0.11	0.092	−0.13	0.083	−0.12	0.083
Apr 2022	−0.01	0.103	−0.07	0.102	−0.09	0.094	−0.08	0.094
N	7,723		7,723		7,723		7,723	
Residual DoF	758		744		743		728	

1*p<0.05; **p<0.01; ***p<0.001

Abbreviations: *CI* Confidence Interval, *SE* Standard Error

All estimates are weighted and inference accounts for the complex survey design

**Table 13:** Differences in wellbeing at wave 2 (conditional on wave 1 wellbeing) by perceived continuing impact of pandemic on wellbeing

	P1		P2		P3		P4	
Characteristic	**Beta** ^*1*^	**SE**	**Beta** ^*1*^	**SE**	**Beta** ^*1*^	**SE**	**Beta** ^*1*^	**SE**
(Intercept)	6.9***	0.041	7.0***	0.093	6.9***	0.089	7.0***	0.104
Negative continuing impact of pandemic on mental wellbeing								
No	—	—	—	—	—	—	—	—
Yes	-1.4***	0.059	-1.3***	0.058	-1.2***	0.057	-1.3***	0.193
Gender								
Male			—	—	—	—	—	—
Female			-0.40***	0.056	-0.41***	0.053	-0.47***	0.063
Non-Binary+			-1.2***	0.184	-0.98***	0.175	-1.2***	0.248
Ethnicity								
White			—	—	—	—	—	—
Mixed			-0.29*	0.118	-0.19	0.120	-0.15	0.144
Black			-0.06	0.073	0.00	0.072	0.05	0.085
Asian			0.10	0.104	0.22*	0.103	0.14	0.127
Other			0.25	0.194	0.33	0.177	0.45*	0.197
Parental Education								
Graduate			—	—	—	—	—	—
Below Graduate			-0.03	0.064	-0.01	0.059	0.01	0.072
No Quals			0.14	0.109	0.22*	0.107	0.30*	0.131
Unknown			-0.10	0.318	0.04	0.342	-0.06	0.418
Housing Tenure								
Own House			—	—	—	—	—	—
Other			-0.23***	0.060	-0.21***	0.059	-0.20**	0.074
IDACI Quintile Group								
1 (High Deprivation)			—	—	—	—	—	—
2			0.19*	0.088	0.16	0.084	0.13	0.102
3			0.22*	0.092	0.19*	0.087	0.13	0.105
4			0.26**	0.091	0.24**	0.087	0.21*	0.105
5 (Low Deprivation)			0.23*	0.094	0.19*	0.088	0.19	0.109
Social Provisions Scale					0.55***	0.029	0.56***	0.039
Negative continuing impact of pandemic on mental wellbeing * Gender								
Yes * Female							0.22	0.117
Yes * Non-Binary+							0.41	0.344
Negative continuing impact of pandemic on mental wellbeing * Ethnicity								
Yes * Mixed							-0.13	0.281
Yes * Black							-0.19	0.168
Yes * Asian							0.28	0.209
Yes * Other							-0.36	0.415
Negative continuing impact of pandemic on mental wellbeing * Parental Education								
Yes * Below Graduate							-0.08	0.124
Yes * No Quals							-0.31	0.222
Yes * Unknown							0.50	0.494
Negative continuing impact of pandemic on mental wellbeing * Housing Tenure								
Yes * Other							-0.02	0.126
Negative continuing impact of pandemic on mental wellbeing * IDACI Quintile Group								
Yes * 2							0.10	0.169
Yes * 3							0.19	0.186
Yes * 4							0.09	0.187
Yes * 5 (Low Deprivation)							-0.01	0.196
Negative continuing impact of pandemic on mental wellbeing * Social Provisions Scale								
Yes * Social Provisions Scale							-0.03	0.058
W2 Month of Survey								
October 2022	—	—	—	—	—	—	—	—
November 2022	-0.04	0.056	-0.09	0.055	-0.09	0.053	-0.09	0.053
December 2022	0.13	0.126	0.09	0.122	0.09	0.116	0.09	0.115
January 2023	0.21	0.271	0.13	0.270	0.10	0.268	0.10	0.268
February 2023	0.47	0.243	0.41	0.256	0.35	0.224	0.36	0.222
March 2023	0.27	0.181	0.21	0.180	0.27	0.182	0.27	0.180
April 2023	0.12	0.208	0.07	0.202	0.18	0.180	0.18	0.180
N	7,723		7,723		7,723		7,723	
Residual DoF	759		745		744		729	

^*1*^*p<0.05; **p<0.01; ***p<0.001

Abbreviations: CI = Confidence Interval, SE = Standard Error

All estimates are weighted and inference accounts for the complex survey design

**Table 14:** Differences in wellbeing at Wave 2 (conditional on Wave 1 wellbeing) by perceived continuing impact of pandemic on wellbeing

	P1		P2		P3		P4	
Characteristic	**Beta** ^*1*^	**SE**	**Beta** ^*1*^	**SE**	**Beta** ^*1*^	**SE**	**Beta** ^*1*^	**SE**
(Intercept)	3.6***	0.121	3.7***	0.150	4.0***	0.158	4.0***	0.163
Negative continuing impact of pandemic on mental wellbeing								
No	—	—	—	—	—	—	—	—
Yes	-0.85***	0.055	-0.81***	0.055	-0.81***	0.055	-0.86***	0.179
Wave 1 Wellbeing	0.48***	0.015	0.46***	0.015	0.42***	0.017	0.42***	0.017
Gender								
Male			—	—	—	—	—	—
Female			-0.19***	0.046	-0.21***	0.046	-0.23***	0.054
Non-Binary+			-0.54**	0.167	-0.53**	0.164	-0.67*	0.269
Ethnicity								
White			—	—	—	—	—	—
Mixed			-0.15	0.095	-0.13	0.097	-0.08	0.127
Black			-0.09	0.060	-0.06	0.061	-0.03	0.072
Asian			0.09	0.089	0.13	0.090	0.08	0.110
Other			0.22	0.172	0.25	0.169	0.37	0.195
Parental Education								
Graduate			—	—	—	—	—	—
Below Graduate			0.03	0.052	0.03	0.051	0.07	0.062
No Quals			0.24**	0.087	0.26**	0.088	0.34***	0.103
Unknown			-0.07	0.264	-0.03	0.273	-0.16	0.332
Housing Tenure								
Own House			—	—	—	—	—	—
Other			-0.18***	0.053	-0.18***	0.053	-0.21**	0.063
IDACI Quintile Group								
1 (High Deprivation)			—	—	—	—	—	—
2			0.12	0.075	0.12	0.075	0.09	0.090
3			0.19*	0.080	0.18*	0.079	0.12	0.094
4			0.15*	0.077	0.15*	0.077	0.17	0.091
5 (Low Deprivation)			0.08	0.078	0.08	0.078	0.06	0.097
Social Provisions Scale					0.19***	0.029	0.20***	0.037
Negative continuing impact of pandemic on mental wellbeing * Gender								
Yes * Female							0.10	0.107
Yes * Non-Binary+							0.28	0.336
Negative continuing impact of pandemic on mental wellbeing * Ethnicity								
Yes * Mixed							-0.14	0.265
Yes * Black							-0.14	0.149
Yes * Asian							0.16	0.189
Yes * Other							-0.33	0.374
Negative continuing impact of pandemic on mental wellbeing * Parental Education								
Yes * Below Graduate							-0.13	0.109
Yes * No Quals							-0.31	0.199
Yes * Unknown							0.64	0.445
Negative continuing impact of pandemic on mental wellbeing * Housing Tenure								
Yes * Other							0.09	0.112
Negative continuing impact of pandemic on mental wellbeing * IDACI Quintile Group								
Yes * 2							0.08	0.155
Yes * 3							0.16	0.166
Yes * 4							-0.06	0.174
Yes * 5 (Low Deprivation)							0.03	0.172
Negative continuing impact of pandemic on mental wellbeing * Social Provisions Scale								
Yes * Social Provisions Scale							-0.02	0.053
W1 Month of Interview								
Sep 2021	—	—	—	—	—	—	—	—
Oct 2021	0.07	0.057	0.05	0.055	0.05	0.055	0.05	0.055
Nov 2021	0.29*	0.113	0.27*	0.116	0.29*	0.114	0.29*	0.115
Dec 2021	0.30*	0.118	0.29*	0.119	0.27*	0.117	0.28*	0.116
Jan 2022	0.15	0.160	0.17	0.150	0.19	0.154	0.19	0.152
Feb 2022	0.32	0.340	0.29	0.334	0.25	0.346	0.25	0.345
Mar 2022	-0.05	0.078	-0.06	0.078	-0.07	0.078	-0.07	0.078
Apr 2022	0.01	0.080	-0.02	0.080	-0.02	0.080	-0.02	0.080
W2 Month of Survey								
October 2022	—	—	—	—	—	—	—	—
November 2022	-0.04	0.049	-0.06	0.049	-0.06	0.049	-0.06	0.049
December 2022	0.19	0.103	0.17	0.102	0.16	0.101	0.16	0.101
January 2023	0.36	0.244	0.32	0.242	0.30	0.244	0.29	0.244
February 2023	0.21	0.217	0.18	0.220	0.18	0.212	0.18	0.211
March 2023	0.32*	0.140	0.29*	0.142	0.30*	0.145	0.30*	0.144
April 2023	0.12	0.180	0.10	0.179	0.13	0.175	0.13	0.174
N	7,723		7,723		7,723		7,723	
Residual DoF	751		737		736		721	

^*1*^*p<0.05; **p<0.01; ***p<0.001

Abbreviations: CI = Confidence Interval, SE = Standard Error

All estimates are weighted and inference accounts for the complex survey design

### Adverse Life Events

**Table 15:** Differences in wellbeing at Wave 1 by number of life events experienced during pandemic

	E1		E2		E3		E4		E5	
Characteristic	**Beta** ^*1*^	**SE**	**Beta** ^*1*^	**SE**	**Beta** ^*1*^	**SE**	**Beta** ^*1*^	**SE**	**Beta** ^*1*^	**SE**
(Intercept)	7.0***	0.064	7.2***	0.107	7.0***	0.101	7.1***	0.099	7.0***	0.143
Adverse Event Tertile Groups										
Fewer	—	—	—	—	—	—	—	—	—	—
Average	-0.58***	0.066	-0.53***	0.066	-0.36***	0.062	-0.30***	0.062	0.15	0.183
More	-1.4***	0.070	-1.3***	0.070	-0.85***	0.068	-0.68***	0.069	-0.70***	0.189
Gender										
Male			—	—	—	—	—	—	—	—
Female			-0.48***	0.056	-0.50***	0.051	-0.41***	0.051	-0.43***	0.081
Non-Binary+			-1.6***	0.204	-1.3***	0.200	-1.0***	0.200	-0.91*	0.409
Ethnicity										
White			—	—	—	—	—	—	—	—
Mixed			-0.23	0.126	-0.11	0.128	-0.12	0.125	-0.12	0.145
Black			0.03	0.086	0.14	0.078	0.12	0.074	0.15	0.087
Asian			-0.03	0.100	0.17	0.091	0.15	0.090	0.11	0.107
Other			0.08	0.220	0.20	0.190	0.20	0.187	0.24	0.218
Parental Education										
Graduate			—	—	—	—	—	—	—	—
Below Graduate			-0.02	0.064	-0.01	0.058	-0.04	0.058	0.03	0.089
No Quals			-0.16	0.126	-0.04	0.116	-0.10	0.114	0.07	0.189
Unknown			-0.01	0.314	0.21	0.337	0.11	0.333	0.32	0.607
Housing Tenure										
Own House			—	—	—	—	—	—	—	—
Other			-0.09	0.067	-0.06	0.063	-0.04	0.062	0.05	0.106
IDACI Quintile Group										
1 (High Deprivation)			—	—	—	—	—	—	—	—
2			0.05	0.091	0.03	0.084	0.06	0.084	0.18	0.141
3			-0.02	0.098	-0.03	0.090	0.00	0.088	0.00	0.158
4			0.17	0.096	0.15	0.	0.17	0.087	0.28*	0.138
5 (Low Deprivation			0.14	0.100	0.12	0.093	0.17	0.091	0.20	0.148
Social Provisions Scale					0.83	0.029	0.81***	0.029	0.88***	0.062
Negative continuing impact of pandemic on mental wellbeing										
No							—	—	—	—
Yes							-0.72***	0.055	-0.71***	0.064
Adverse Event Tertile Groups * Gender										
Average * Female									-0.08	0.111
More * Female									0.14	0.124
Average * Non-Binary+									-0.14	0.572
More * Non-Binary+									-0.16	0.464
Ethnicity * Negative continuing impact of pandemic on mental wellbeing										
Mixed * Yes									-0.03	0.211
Black * Yes									-0.12	0.151
Asian * Yes									0.15	0.171
Other * Yes									-0.16	0.411
Adverse Event Tertile Groups * Parental Education										
Average * Below Graduate									0.00	0.130
More * Below Graduate									-0.20	0.136
Average * No Quals									-0.26	0.234
More * No Quals									-0.31	0.283
Average * Unknown									-0.93	0.747
More * Unknown									0.	0.749
Adverse Event Tertile Groups * Housing Tenure										
Average *									-0.	0.147
More * Other									-0.04	0.150
Adverse Event Tertile Groups * IDACI Quintile Group										
Average * 2									-0.36	0.197
More * 2									-0.12	0.194
Average * 3									-0.25	0.210
More * 3									0.18	0.208
Average * 4									-0.57**	0.192
More * 4									0.12	0.193
Average * 5 (Low Deprivation)									-0.25	0.202
More * 5 (Low Deprivation)									0.07	0.211
Adverse Event Tertile Groups * Social Provisions Scale										
Average * Social Provisions Scale									-0.03	0.075
More * Social Provisions Scale									-0.14	0.072
W1 Month of Interview										
Sep 2021	—	—	—	—	—	—	—	—	—	—
Oct 2021	0.10	0.065	0.07	0.065	0.02	0.059	0.02	0.059	0.03	0.059
Nov 2021	0.42*	0.186	0.37*	0.183	0.39*	0.168	0.35*	0.168	0.36*	0.165
Dec 2021	0.26*	0.126	0.24	0.125	0.12	0.113	0.	0.111	0.10	0.109
Jan 2022	0.45	0.262	0.44	0.246	0.45	0.253	0.43	0.255	0.42	0.255
Feb 2022	-0.24	0.263	-0.38	0.250	-0.50*	0.247	-0.60*	0.238	-0.60*	0.240
Mar 2022	-0.11	0.094	-0.13	0.092	-0.15	0.084	-0.13	0.083	-0.13	0.
Apr 2022	-0.06	0.099	-0.11	0.098	-0.11	0.093	-0.11	0.092	-0.10	0.091
N	7,723		7,		7,723		7,723		7,723	
Residual DoF	757		743		742		741		715	

^*1*^*p<0.05; **p<0.01; ***p<0.001

Abbreviations: *CI* Confidence Interval, *SE* Standard Error

All estimates are weighted and inference accounts for the complex survey design

**Table 16:** Differences in wellbeing at Wave 2 by number of life events experienced during pandemic

	E1		E2		E3		E4		E5	
Characteristic	**Beta** ^*1*^	**SE**	**Beta** ^*1*^	**SE**	**Beta** ^*1*^	**SE**	**Beta** ^*1*^	**SE**	**Beta** ^*1*^	**SE**
(Intercept)	7.0***	0.049	7.2***	0.098	7.0***	0.095	7.2***	0.092	7.2***	0.123
Adverse Event Tertile Groups										
Fewer	—	—	—	—	—	—	—	—	—	—
Average	-0.55***	0.064	-0.50***	0.063	-0.39***	0.063	-0.31***	0.061	-0.32	0.190
More	-1.3***	0.066	-1.1***	0.066	-0.88***	0.067	-0.64***	0.067	-0.61***	0.182
Gender										
Male			—	—	—	—	—	—	—	—
Female			-0.48***	0.054	-0.49***	0.052	-0.35***	0.053	-0.35***	0.080
Non-Binary+			-1.4***	0.174	-1.2***	0.167	-0.92***	0.171	-0.92**	0.346
Ethnicity										
White			—	—	—	—	—	—	—	—
Mixed			-0.24	0.124	-0.16	0.126	-0.18	0.120	-0.15	0.139
Black			-0.06	0.078	0.01	0.075	-0.02	0.072	0.02	0.083
Asian			0.09	0.101	0.22*	0.100	0.18	0.099	0.09	0.121
Other			0.23	0.202	0.31	0.186	0.32	0.177	0.43*	0.195
Parental Education										
Graduate			—	—	—	—	—	—	—	—
Below Graduate			0.03	0.064	0.04	0.060	-0.01	0.059	-0.05	0.090
No Quals			0.22*	0.112	0.30**	0.109	0.20	0.106	0.37*	0.160
Unknown			0.02	0.325	0.15	0.347	0.01	0.345	-0.18	0.338
Housing Tenure										
Own House			—	—	—	—	—	—	—	—
Other			-0.24***	0.062	-0.22***	0.061	-0.18**	0.059	-0.16	0.096
IDACI Quintile Group										
1 (High Deprivation)			—	—	—	—	—	—	—	—
2			0.10	0.086	0.09	0.083	0.13	0.083	0.16	0.126
3			0.13	0.093	0.11	0.088	0.17*	0.086	0.16	0.135
4			0.22*	0.093	0.20*	0.090	0.23**	0.087	0.17	0.126
5 (Low Deprivation)			0.09	0.095	0.07	0.091	0.14	0.087	0.06	0.137
Social Provisions Scale					0.53***	0.030	0.50***	0.030	0.57***	0.063
Negative continuing impact of pandemic on mental wellbeing										
No							—	—	—	—
Yes							-1.1***	0.057	-1.0***	0.065
Adverse Event Tertile Groups * Gender										
Average * Female									-0.05	0.124
More * Female									0.01	0.131
Average * Non-Binary+									0.37	0.472
More * Non-Binary+									-0.23	0.389
Ethnicity * Negative continuing impact of pandemic on mental wellbeing										
Mixed * Yes									-0.	0.281
Black * Yes									-0.17	0.150
Asian * Yes									0.31	0.192
Other * Yes									-0.	0.410
Adverse Event Tertile Groups * Parental Education										
Average * Below Graduate									0.09	0.132
More * Below Graduate									0.	0.139
Average * No Quals									-0.	0.229
More * No Quals									-0.39	0.255
Average * Unknown									0.17	0.805
More * Unknown									0.38	0.599
Adverse Event Tertile Groups * Housing Tenure										
Average * Other									0.10	0.155
More * Other									-0.15	0.143
Adverse Event Tertile Groups * IDACI Quintile Group										
Average * 2									-0.11	0.196
More * 2									-0.05	0.198
Average * 3									-0.10	0.204
More * 3									0.09	0.191
Average * 4									0.00	0.197
More * 4									0.13	0.183
Average * 5 (Low Deprivation)									0.11	0.216
More * 5 (Low Deprivation)									0.13	0.208
Adverse Event Tertile Groups * Social Provisions Scale										
Average * Social Provisions Scale									-0.06	0.082
More * Social Provisions Scale									-0.11	0.072
W2 Month of Survey										
October 2022	—	—	—	—	—	—	—	—	—	—
November 2022	-0.05	0.057	-0.10	0.056	-0.10	0.055	-0.10	0.053	-0.09	0.053
December 2022	0.19	0.124	0.15	0.118	0.15	0.114	0.09	0.113	0.10	0.113
January 2023	0.35	0.310	0.25	0.301	0.21	0.296	0.12	0.279	0.10	0.273
February 2023	0.60*	0.234	0.53*	0.246	0.48*	0.221	0.35	0.224	0.34	0.221
March 2023	0.39*	0.186	0.31	0.187	0.35	0.185	0.28	0.185	0.27	0.183
April 2023	0.17	0.216	0.13	0.210	0.23	0.188	0.19	0.184	0.17	0.185
N	7,723		7,723		7,723		7,723		7,723	
Residual DoF	758		744		743		742		716	

^*1*^*p<0.05; **p<0.01; ***p<0.001

Abbreviations:*CI*Confidence Interval,*SE* Standard Error

All estimates are weighted and inference accounts for the complex survey design

**Table 17:** Differences in wellbeing at Wave 2 (conditional on Wave 1 wellbeing) by number of life events experienced during pandemic

	E1		E2		E3		E4		E5	
Characteristic	**Beta** ^*1*^	**SE**	**Beta** ^*1*^	**SE**	**Beta** ^*1*^	**SE**	**Beta** ^*1*^	**SE**	**Beta** ^*1*^	**SE**
(Intercept)	3.5***	0.126	3.7***	0.154	4.2***	0.163	4.2***	0.163	4.3***	0.178
Adverse Event Tertile Groups										
Fewer	—	—	—	—	—	—	—	—	—	—
Average	-0.26***	0.056	-0.25***	0.056	-0.19***	0.054	-0.19***	0.054	-0.37*	0.167
More	-0.59***	0.060	-0.54***	0.060	-0.36***	0.060	-0.36***	0.060	-0.33	0.171
Wave 1 Wellbeing	0.49***	0.015	0.48***	0.015	0.41***	0.017	0.41***	0.017	0.41***	0.017
Gender										
Male			—	—	—	—	—	—	—	—
Female			-0.24***	0.045	-0.18***	0.046	-0.18***	0.046	-0.17*	0.070
Non-Binary+			-0.69***	0.162	-0.50**	0.162	-0.50**	0.162	-0.52	0.315
Ethnicity										
White			—	—	—	—	—	—	—	—
Mixed			-0.12	0.098	-0.13	0.098	-0.13	0.098	-0.10	0.126
Black			-0.08	0.061	-0.07	0.061	-0.07	0.061	-0.04	0.071
Asian			0.09	0.088	0.11	0.089	0.11	0.089	0.03	0.106
Other			0.21	0.177	0.25	0.169	0.25	0.169	0.34	0.194
Parental Education										
Graduate			—	—	—	—	—	—	—	—
Below Graduate			0.06	0.053	0.03	0.051	0.03	0.051	-0.03	0.077
No Quals			0.30***	0.088	0.25**	0.088	0.25**	0.088	0.34*	0.135
Unknown			0.01	0.263	-0.04	0.276	-0.04	0.276	-0.30	0.245
Housing Tenure										
Own House			—	—	—	—	—	—	—	—
Other			-0.20***	0.054	-0.17**	0.053	-0.17**	0.053	-0.18*	0.081
IDACI Quintile Group										
1 (High Deprivation)			—	—	—	—	—	—	—	—
2			0.07	0.074	0.10	0.075	0.10	0.075	0.08	0.111
3			0.13	0.081	0.17*	0.079	0.17*	0.079	0.16	0.117
4			0.12	0.079	0.15	0.077	0.15	0.077	0.05	0.114
5 (Low Deprivation)			0.00	0.079	0.05	0.078	0.05	0.078	-0.03	0.119
Social Provisions Scale					0.17***	0.029	0.17***	0.029	0.21***	0.053
Negative continuing impact of pandemic on mental wellbeing										
No					—	—	—	—	—	—
Yes					-0.76***	0.055	-0.76***	0.055	-0.74***	0.062
Adverse Event Tertile Groups * Gender										
Average * Female									-0.02	0.111
More * Female									-0.04	0.121
Average * Non-Binary+									0.37	0.434
More * Non-Binary+									-0.20	0.359
Ethnicity * Negative continuing impact of pandemic on mental wellbeing										
Mixed * Yes									-0.12	0.266
Black * Yes									-0.13	0.135
Asian * Yes									0.23	0.174
Other * Yes									-0.35	0.374
Adverse Event Tertile Groups * Parental Education										
Average * Below Graduate									0.09	0.118
More * Below Graduate									0.11	0.123
Average * No Quals									-0.04	0.207
More * No Quals									-0.24	0.208
Average * Unknown									0.47	0.647
More * Unknown									0.20	0.440
Adverse Event Tertile Groups * Housing Tenure										
Average * Other									0.22	0.136
More * Other									-0.14	0.126
Adverse Event Tertile Groups * IDACI Quintile Group										
Average * 2									0.03	0.174
More * 2									0.02	0.183
Average * 3									-0.01	0.176
More * 3									0.03	0.174
Average * 4									0.23	0.177
More * 4									0.07	0.170
Average * 5 (Low Deprivation)									0.18	0.193
More * 5 (Low Deprivation)									0.08	0.185
Adverse Event Tertile Groups * Social Provisions Scale										
Average * Social Provisions Scale									-0.04	0.073
More * Social Provisions Scale									-0.05	0.061
W1 Month of Interview										
Sep 2021	—	—	—	—	—	—	—	—	—	—
Oct 2021	0.06	0.058	0.05	0.056	0.05	0.055	0.05	0.055	0.04	0.055
Nov 2021	0.32**	0.114	0.32**	0.118	0.31**	0.116	0.31**	0.116	0.30*	0.118
Dec 2021	0.31**	0.111	0.31**	0.112	0.27*	0.114	0.27*	0.114	0.26*	0.113
Jan 2022	0.13	0.166	0.17	0.155	0.19	0.156	0.19	0.156	0.16	0.159
Feb 2022	0.44	0.347	0.41	0.339	0.26	0.348	0.26	0.348	0.27	0.350
Mar 2022	-0.06	0.078	-0.08	0.078	-0.07	0.077	-0.07	0.077	-0.08	0.077
Apr 2022	-0.02	0.080	-0.04	0.080	-0.04	0.080	-0.04	0.080	-0.04	0.079
W2 Month of Survey										
October 2022	—	—	—	—	—	—	—	—	—	—
November 2022	-0.04	0.049	-0.06	0.049	-0.06	0.049	-0.06	0.049	-0.06	0.048
December 2022	0.23*	0.102	0.21*	0.101	0.16	0.100	0.16	0.100	0.16	0.099
January 2023	0.45	0.269	0.40	0.264	0.30	0.253	0.30	0.253	0.29	0.249
February 2023	0.29	0.212	0.25	0.215	0.18	0.213	0.18	0.213	0.17	0.210
March 2023	0.39**	0.144	0.35*	0.146	0.30*	0.148	0.30*	0.148	0.30*	0.147
April 2023	0.15	0.185	0.13	0.183	0.14	0.177	0.14	0.177	0.13	0.177
N	7,723		7,723		7,723		7,723		7,723	
Residual DoF	750		736		734		734		708	

^*1*^*p<0.05; **p<0.01; ***p<0.001

Abbreviations: *CI * Confidence Interval, *SE* Standard Error

All estimates are weighted and inference accounts for the complex survey design

## Appendix: Supplementary Results

### Demographic Differences in Wellbeing

Across Waves 1 and 2 (panels 1 and 2 of Fig. 9), the only significant unconditional differences in young people’s wellbeing are between those classified as White and those classified as of Mixed ethnicity. No difference emerges when other demographic characteristics are included in model L4. However, these lower levels among those with Mixed ethnicity are explained by differences in social support, while, conversely, including this covariate reveals a significant difference in wellbeing between those classified as White and those classified as Black and Asian in model L6. This latter finding implies that if Black and Asian young people reported the same scores on the social provisions scale as White young people, their wellbeing scores would be higher. This difference is only present at Wave 2 for those with an Asian ethnicity, and is not present for any group when we are looking at Wave 2 wellbeing having adjusted for wellbeing at Wave 1.

The differences that emerged for Black and Asian young people in model L6 appear slightly attenuated by differences in adverse life events (0.16 for Black young people and 0.18 for Asian young people), but not by much and the estimates in L6 and L8 are not statistically significant from one another.

There is evidence of a gradient in wellbeing by SES, with a roughly linear pattern across SES quintile groups at both Waves 1 and 2. However, the differences are only significant in the unconditional model (L3) once we reach the top two quintile groups, compared to the bottom. The overall difference between the top and bottom quintile groups is 0.3 points at Wave 1 and a bit larger (0.33 points) at Wave 2.

There is essentially no difference when gender and ethnicity are included in model L4, but some of the SES gradient is attenuated by differences in social support when these are included in model L6. For Wave 1, the difference between the bottom and the second-highest quintile groups becomes statistically insignificant, although this is not the case for differences at Wave 2, given their slightly larger overall magnitude. The conditional difference between the top and bottom quintile groups is 0.21 points at Wave 1 and, again, a bit larger (0.24 points) at Wave 2.

Ultimately, even these differences between the top and bottom SES quintile groups are attenuated to statistical insignificance when we adjust for experiences of adverse life events in model L8 (although we should note that the differences in coefficients between models L6 and L8 are not themselves statistically significant). This is the case for both Waves 1 and 2, and for Wave 2 after adjusting for Wave 1 wellbeing. It would seem that, between them, we can account for much of the socioeconomic variation in wellbeing with social support and experiences of adverse life events — although it is important to note that this is not the same as saying that socioeconomic inequalities in wellbeing are unimportant, especially as socioeconomic status is likely to affect levels of social support and adverse life events.

We explore the potential for intersectional differences between the demographic characteristics using model L5, but find little evidence of any clear or consistent patterns of this type. Similarly, we allow for moderation of the importance of social support by demographic characteristics in model L7, but find little evidence of this either.

### Perceived Continuing Impact of the Pandemic On Wellbeing

Building on analysis in Sect. 4.2, we also explore whether the difference in wellbeing associated with having a negative perception of the impact of the pandemic on wellbeing is moderated by social provisions. We do so using Model P4 reported in Table 12 (for Wave 1), Table 13 (for Wave 2) and Table 14 (for Wave 2 adjusting for Wave 1) which includes an interaction term between these two factors. Since interaction terms can be complicated to interpret, we illustrate the results using predicted probabilities from the models (with all other characteristics held constant at means or modal values) plotted in Fig. 12.

While we find associations with each of these two measures (approximately 1 point lower wellbeing among those with a negative perceived impact of the pandemic, as discussed in Sect. 4.2; a positive gradient associated with higher levels of social provisions), we find no evidence that the difference in wellbeing predicted by a negative perceived ongoing impact of the pandemic on wellbeing depends on the level of social provisions. Put another way, we expect the same change in wellbeing associated with a negative perceived impact of the pandemic on wellbeing regardless of the level of social provisions reported by the young person. This is true at both Waves 1 and 2, and for Wave 2 after adjusting for Wave 1 wellbeing.

## Appendix: Multiple Imputation

**Table 18:** Regression of wellbeing at Wave 1 on perceived negative impact of COVID-19 on mental wellbeing

Characteristic	Beta^*1*^	SE
(Intercept)	3.3***	0.133
Gender		
Male	—	—
Female	-0.48***	0.051
Non-Binary+	-1.2***	0.179
Ethnicity		
White	—	—
Mixed	-0.10	0.113
Black	0.15*	0.072
Asian	0.17*	0.079
Other	0.18	0.175
SES Quintile Group		
Q1 (Low)	—	—
Q2	-0.10	0.082
Q3	0.04	0.080
Q4	-0.01	0.076
Q5 (High)	0.07	0.080
Social Provisions Scale	0.64***	0.021
Adverse Event Index	-0.50***	0.036
Wave 1 Survey Month		
Sep 2021	—	—
Oct 2021	0.03	0.057
Nov 2021	0.38*	0.156
Dec 2021	0.10	0.107
Jan 2022	0.33	0.246
Feb 2022	-0.45*	0.209
Mar 2022	-0.12	0.080
Apr 2022	-0.08	0.088
N	9,307	

^*1*^*p<0.05; **p<0.01; ***p<0.001

Abbreviations: CI = Confidence Interval, SE = Standard Error

All estimates are weighted and inference accounts for the complex survey design. Minimum residual degrees of freedom in any of the 10 imputations = 748

**Table 19:** Regression of wellbeing at Wave 2 on perceived negative impact of COVID-19 on mental wellbeing

Characteristic	Beta^*1*^	SE
(Intercept)	4.8***	0.128
Negative continuing impact of pandemic on mental wellbeing		
No	—	—
Yes	-1.0***	0.055
Gender		
Male	—	—
Female	-0.32***	0.049
Non-Binary+	-0.96***	0.165
Ethnicity		
White	—	—
Mixed	-0.17	0.108
Black	0.08	0.066
Asian	0.05	0.087
Other	0.30	0.161
SES Quintile Group		
Q1 (Low)	—	—
Q2	0.09	0.076
Q3	0.07	0.075
Q4	0.15	0.077
Q5 (High)	0.26**	0.085
Social Provisions Scale	0.39***	0.021
Adverse Event Index	-0.39***	0.035
Wave 2 Survey Month		
October 2022	—	—
November 2022	-0.11*	0.052
December 2022	0.09	0.103
January 2023	0.24	0.288
February 2023	0.35	0.207
March 2023	0.41*	0.183
April 2023	0.02	0.176
N	9,307	

^*1*^*p<0.05; **p<0.01; ***p<0.001

Abbreviations: *CI* Confidence Interval, *SE* Standard Error

All estimates are weighted and inference accounts for the complex survey design. Minimum residual degrees of freedom in any of the 10 imputations = 748

**Table 20:** Regression of wellbeing at Wave 2 (adjusted for wellbeing at Wave 1) on perceived negative impact of COVID-19 on mental wellbeing

Characteristic	Beta^*1*^	SE
(Intercept)	3.3***	0.133
Wave 1 Wellbeing	0.41***	0.016
Negative continuing impact of pandemic on mental wellbeing		
No	—	—
Yes	-0.75***	0.052
Gender		
Male	—	—
Female	-0.16***	0.043
Non-Binary+	-0.56***	0.161
Ethnicity		
White	—	—
Mixed	-0.12	0.090
Black	0.03	0.057
Asian	-0.03	0.079
Other	0.23	0.148
SES Quintile Group		
Q1 (Low)	—	—
Q2	0.14*	0.069
Q3	0.04	0.067
Q4	0.14*	0.070
Q5 (High)	0.17*	0.072
Social Provisions Scale	0.13***	0.021
Adverse Event Index	-0.22***	0.032
Wave 1 Survey Month		
Sep 2021	—	—
Oct 2021	0.01	0.052
Nov 2021	0.25*	0.123
Dec 2021	0.36**	0.113
Jan 2022	0.19	0.175
Feb 2022	0.22	0.284
Mar 2022	-0.03	0.069
Apr 2022	-0.03	0.075
Wave 2 Survey Month		
October 2022	—	—
November 2022	-0.08	0.047
December 2022	0.13	0.094
January 2023	0.40	0.265
February 2023	0.20	0.201
March 2023	0.44**	0.151
April 2023	0.01	0.175
N	9,307	

^*1*^*p<0.05; **p<0.01; ***p<0.001

Abbreviations: *CI * Confidence Interval, *SE* Standard Error

All estimates are weighted and inference accounts for the complex survey design. Minimum residual degrees of freedom in any of the 10 imputations = 740

**Table 21:** Regression of wellbeing at Wave 1 on perceived negative impact of COVID-19 on mental wellbeing

Characteristic	Beta^*1*^	SE
(Intercept)	3.5***	0.169
Negative continuing impact of pandemic on mental wellbeing		
No	—	—
Yes	-0.84***	0.052
Gender		
Male	—	—
Female	-0.45***	0.050
Non-Binary+	-1.1***	0.184
Ethnicity		
White	—	—
Mixed	-0.11	0.113
Black	0.14	0.072
Asian	0.18*	0.080
Other	0.18	0.171
Parental Education	-0.02	0.042
Housing Tenure	-0.05	0.057
IDACI Quintile Group		
1 (High Deprivation)	—	—
2	0.07	0.077
3	0.03	0.084
4	0.17*	0.082
5 (Low Deprivation)	0.20*	0.088
Social Provisions Scale	0.67***	0.020
Wave 1 Survey Month		
Sep 2021	—	—
Oct 2021	0.02	0.057
Nov 2021	0.33*	0.160
Dec 2021	0.10	0.110
Jan 2022	0.	0.249
Feb 2022	-0.54**	0.199
Mar 2022	-0.12	0.080
Apr 2022	-0.06	0.087
N	9,307	

^*1*^*p<0.05; **p<0.01; ***p<0.001

Abbreviations: *CI* Confidence Interval, *SE* Standard Error

All estimates are weighted and inference accounts for the complex survey design. Minimum residual degrees of freedom in any of the 10 imputations = 746

**Table 22:** Regression of wellbeing at Wave 2 on perceived negative impact of COVID-19 on mental wellbeing

Characteristic	Beta^*1*^	SE
(Intercept)	4.8***	0.169
Negative continuing impact of pandemic on mental wellbeing		
No		—
Yes	-1.2***	0.054
Gender		
Male	—	—
Female	-0.38***	0.050
Non-Binary+	-1.1***	0.167
Ethnicity		
White	—	—
Mixed	-0.16	0.110
Black	0.07	0.068
Asian	0.10	0.092
Other	0.31	0.163
Parental Education	0.04	0.046
Housing Tenure	-0.17**	0.058
IDACI Quintile Group		
1 (High Deprivation)	—	—
2	0.16*	0.076
3	0.14	0.081
4	0.19*	0.081
5 (Low Deprivation)	0.21*	0.087
Social Provisions Scale	0.43***	0.021
Wave 2 Survey Month		
October 2022	—	—
November 2022	-0.10	0.052
December 2022	0.08	0.107
January 2023	0.23	0.277
February 2023	0.35	0.204
March 2023	0.40*	0.181
April 2023	0.00	0.173
N	9,307	

^*1*^*p<0.05; **p<0.01; ***p<0.001

Abbreviations: *CI* Confidence Interval, *SE* Standard Error

All estimates are weighted and inference accounts for the complex survey design. Minimum residual degrees of freedom in any of the 10 imputations = 747

**Table 23:** Regression of wellbeing at Wave 2 (adjusted for wellbeing at Wave 1) on perceived negative impact of COVID-19 on mental wellbeing

Characteristic	Beta^*1*^	SE
(Intercept)	3.3***	0.170
Wave 1 Wellbeing	0.42***	0.015
Negative continuing impact of pandemic on mental wellbeing		
No	—	—
Yes	-0.81***	0.051
Gender		
Male	—	—
Female	-0.19***	0.043
Non-Binary+	-0.61***	0.162
Ethnicity		
White	—	—
Mixed	-0.11	0.090
Black	0.01	0.058
Asian	0.01	0.082
Other	0.24	0.150
Parental Education	0.06	0.039
Housing Tenure	-0.15**	0.052
IDACI Quintile Group		
1 (High Deprivation)	—	—
2	0.12	0.066
3	0.12	0.075
4	0.11	0.072
5 (Low Deprivation)	0.10	0.074
Social Provisions Scale	0.15***	0.021
Wave 1 Survey Month		
Sep 2021	—	—
Oct 2021	0.01	0.052
Nov 2021	0.24*	0.121
Dec 2021	0.36**	0.114
Jan 2022	0.21	0.172
Feb 2022	0.22	0.285
Mar 2022	-0.04	0.070
Apr 2022	-0.02	0.076
Wave 2 Survey Month		
October 2022	—	—
November 2022	-0.08	0.047
December 2022	0.12	0.096
January 2023	0.39	0.255
February 2023	0.18	0.197
March 2023	0.43**	0.150
April 2023	-0.01	0.172
N	9,307	

^*1*^*p<0.05; **p<0.01; ***p<0.001

Abbreviations: *CI * Confidence Interval, *SE* Standard Error

All estimates are weighted and inference accounts for the complex survey design. Minimum residual degrees of freedom in any of the 10 imputations = 739

**Table 24:** Regression of wellbeing at Wave 1 on adverse life events during the pandemic

Characteristic	Beta^*1*^	SE
(Intercept)	4.0***	0.179
Adverse Event Tertile Groups		
Low	—	—
Medium	-0.34***	0.060
High	-0.75***	0.067
Negative continuing impact of pandemic on mental wellbeing		
No	—	—
Yes	-0.70***	0.052
Gender		
Male	—	—
Female	-0.39***	0.050
Non-Binary+	-1.0***	0.178
Ethnicity		
White	—	—
Mixed	-0.10	0.113
Black	0.11	0.071
Asian	0.14	0.078
Other	0.17	0.172
Parental Education	-0.02	0.042
Housing Tenure	-0.03	0.056
IDACI Quintile Group		
1 (High Deprivation)	—	
2	0.05	0.076
3	0.01	0.084
4	0.17*	0.081
5 (Low Deprivation)	0.15	0.086
Social Provisions Scale	0.63***	0.021
Wave 1 Survey Month		
Sep 2021	—	—
Oct 2021	0.02	0.056
Nov 2021	0.36*	0.162
Dec 2021	0.08	0.107
Jan 2022	0.33	0.248
Feb 2022	-0.53**	0.202
Mar 2022	-0.12	0.080
Apr 2022	-0.09	0.086
N	9,307	

^*1*^*p<0.05; **p<0.01; ***p<0.001

Abbreviations: *CI* Confidence Interval,* SE* Standard Error

All estimates are weighted and inference accounts for the complex survey design. Minimum residual degrees of freedom in any of the 10 imputations = 744

**Table 25:** Regression of wellbeing at Wave 2 on adverse life events during the pandemic

Characteristic	Beta^*1*^	SE
(Intercept)	5.3***	0.176
Adverse Event Tertile Groups		
Low	—	—
Medium	-0.34***	0.060
High	-0.67***	0.068
Negative continuing impact of pandemic on mental wellbeing		
No	—	—
Yes	-1.0***	0.054
Gender		
Male	—	—
Female	-0.33***	0.050
Non-Binary+	-0.99***	0.163
Ethnicity		
White	—	—
Mixed	-0.16	0.110
Black	0.05	0.067
Asian	0.07	0.089
Other	0.30	0.163
Parental Education	0.04	0.046
Housing Tenure	-0.15**	0.057
IDACI Quintile Group		
1 (High Deprivation)	—	—
2	0.13	0.074
3	0.12	0.080
4	0.19*	0.080
5 (Low Deprivation)	0.17*	0.085
Social Provisions Scale	0.40***	0.022
Wave 2 Survey Month		
October 2022	—	—
November 2022	-0.11*	0.052
December 2022	0.08	0.105
January 2023	0.25	0.292
February 2023	0.35	0.204
March 2023	0.42*	0.184
April 2023	0.01	0.175
N	9,307	

^*1*^*p<0.05; **p<0.01; ***p<0.001

Abbreviations: *CI* Confidence Interval, *SE*Standard Error

All estimates are weighted and inference accounts for the complex survey design. Minimum residual degrees of freedom in any of the 10 imputations = 745

**Table 26:** Regression of wellbeing at Wave 2 (adjusted for wellbeing at Wave 1) on adverse life events during the pandemic

Characteristic	Beta^*1*^	SE
(Intercept)	3.6***	0.179
Wave 1 Wellbeing	0.41***	0.016
Adverse Event Tertile Groups		
Low	—	—
Medium	-0.20***	0.055
High	-0.36***	0.060
Negative continuing impact of pandemic on mental wellbeing		
No	—	—
Yes	-0.75***	0.051
Gender		
Male	—	—
Female	-0.17***	0.043
Non-Binary+	-0.59***	0.160
Ethnicity		
White	—	—
Mixed	-0.11	0.090
Black	0.00	0.058
Asian	-0.01	0.081
Other	0.24	0.150
Parental Education	0.05	0.039
Housing Tenure	-0.14**	0.052
IDACI Quintile Group		
1 (High Deprivation)	—	—
2	0.10	0.066
3	0.11	0.075
4	0.11	0.072
5 (Low Deprivation)	0.09	0.074
Social Provisions Scale	0.14***	0.021
Wave 1 Survey Month		
Sep 2021	—	—
Oct 2021	0.01	0.052
Nov 2021	0.26*	0.125
Dec 2021	0.36**	0.113
Jan 2022	0.20	0.173
Feb 2022	0.22	0.287
Mar 2022	-0.04	0.069
Apr 2022	-0.04	0.075
Wave 2 Survey Month		
October 2022	—	—
November 2022	-0.08	0.047
December 2022	0.12	0.095
January 2023	0.40	0.266
February 2023	0.19	0.198
March 2023	0.44**	0.152
April 2023	0.00	0.174
N	9,307	

^*1*^*p<0.05; **p<0.01; ***p<0.001

Abbreviations: *CI* Confidence Interval, *SE* Standard Error

All estimates are weighted and inference accounts for the complex survey design. Minimum residual degrees of freedom in any of the 10 imputations = 737
